# Chronic mild stress paradigm as a rat model of depression: facts, artifacts, and future perspectives

**DOI:** 10.1007/s00213-021-05982-w

**Published:** 2022-01-24

**Authors:** Tatyana Strekalova, Yanzhi Liu, Daniel Kiselev, Sharafuddin Khairuddin, Jennifer Lok Yu Chiu, Justin Lam, Ying-Shing Chan, Dmitrii Pavlov, Andrey Proshin, Klaus-Peter Lesch, Daniel C. Anthony, Lee Wei Lim

**Affiliations:** 1 Department of Psychiatry and Neuropsychology, Maastricht University, Maastricht, Netherlands; 2 Department of Normal Physiology and Laboratory of Psychiatric Neurobiology, Sechenov First Moscow State Medical University, Moscow, Russia; 3 Division of Molecular Psychiatry, Center of Mental Health, University of Würzburg, Würzburg, Germany; 4 School of Biomedical Sciences, Li Ka Shing Faculty of Medicine, The University of Hong Kong, Pok Fu Lam, Hong Kong; 5 Institute of General Pathology and Pathophysiology, RAS, Moscow, Russia; 6 P.K. Anokhin Research Institute of Normal Physiology, Moscow, Russia; 7 Department of Pharmacology, Oxford University, Oxford, UK

**Keywords:** Chronic mild stress (CMS), Depression, Sucrose test, Anhedonia, Forced swimming, Open field, Inter-individual variability, Stress resilience, Rat

## Abstract

**Rationale:**

The chronic mild stress (CMS) paradigm was first described almost 40 years ago and has become a widely used model in the search for antidepressant drugs for major depression disorder (MDD). It has resulted in the publication of almost 1700 studies in rats alone. Under the original CMS procedure, the expression of an anhedonic response, a key symptom of depression, was seen as an essential feature of both the model and a depressive state. The prolonged exposure of rodents to unpredictable/uncontrollable mild stressors leads to a reduction in the intake of palatable liquids, behavioral despair, locomotor inhibition, anxiety-like changes, and vegetative (somatic) abnormalities. Many of the CMS studies do not report these patterns of behaviors, and they often fail to include consistent molecular, neuroanatomical, and physiological phenotypes of CMS-exposed animals.

**Objectives:**

To critically review the CMS studies in rats so that conceptual and methodological flaws can be avoided in future studies.

**Results:**

Analysis of the literature supports the validity of the CMS model and its impact on the field. However, further improvements could be achieved by (i) the stratification of animals into ‘resilient’ and ‘susceptible’ cohorts within the CMS animals, (ii) the use of more refined protocols in the sucrose test to mitigate physiological and physical artifacts, and (iii) the systematic evaluation of the non-specific effects of CMS and implementation of appropriate adjustments within the behavioral tests.

**Conclusions:**

We propose methodological revisions and the use of more advanced behavioral tests to refine the rat CMS paradigm, which offers a valuable tool for developing new antidepressant medications.

**Supplementary Information:**

The online version contains supplementary material available at 10.1007/s00213-021-05982-w.

## Introduction

Major depressive disorder (MDD) is a common psychiatric illness that has an enormous impact on quality of life. In 2012, the World Health Organization (WHO) described MDD as a ‘global crisis’. Almost a decade later, it remains a leading contributor to the global burden of disease. Moreover, the treatment of MDD continues to pose significant challenges for clinicians. In the United States, depression has a prevalence of 10%, and up to one-in-five individuals will experience MDD over the course of a lifetime (Hasin et al. 2018; Gauthier et al. 2019). Furthermore, the COVID-19 outbreak and associated social distancing rules have increased the prevalence of MDD (Chaturvedi 2020; Wind et al. 2020). Thus, the incidence of mental disorders, including MDD, is likely to rise, which will impact not only on the individuals affected, but also their relatives, caregivers, and the wider community (Wind et al. 2020). Consequently, the need to identify new effective therapy is urgently required, and this requires the use of clinically relevant preclinical models and appropriate outcome measures.

**Fig. 1:**
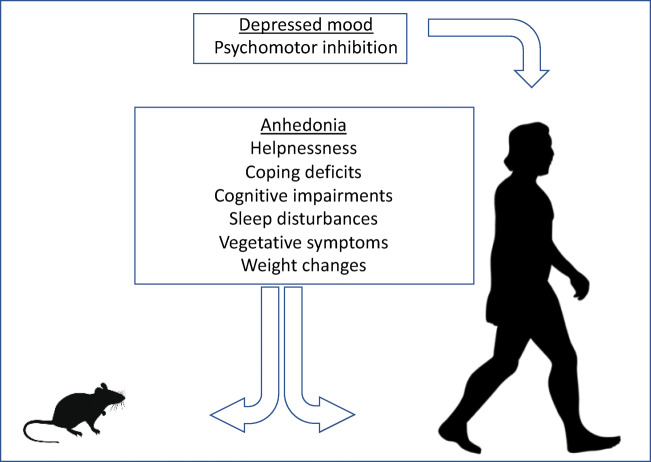
Symptoms of major depression in translational aspect. Major depression is defined by the occurrence of at least one core symptom (underlined) lasting minimally two weeks that is typically accompanied by a number of subsidiary symptoms. Some of these symptoms are purely human phenomena though others can be recapitulated in laboratory rodents, including rats (*see the text*)

The Diagnostic and Statistical Manual, 5th Edition (DSMV), defines MDD as the ‘presence of at least one core symptom, lasting for a minimum of two weeks that is typically accompanied by subsidiary symptoms’. Anhedonia, a decreased ability to experience pleasure, together with a persistently low mood, is commonly regarded as a key symptom of clinical depression (Hamilton 1967; Klein 1974). MDD is also often associated with psychomotor inhibition, vegetative (somatic) symptoms, cognitive abnormalities, changes in appetite and body weight (Kessler et al. 2005; Kessler and Bromet 2013; Rizvi et al. 2016), as well as sleep disturbances (Baglioni et al. 2011). Some of these symptoms can be reproduced in animals (Fig. 1).

Despite a variety of therapeutic regimens available for depression, many of them appear to be effective in only about a half of patients, and up to 35% of MDD cases remain refractory to treatment (Munos 2009; Pigott et al. 2010; Insel and Sahakian 2012; Moeler 2017; Dubovsky 2018; Safer and Zito 2019). Drug resistance in the treatment of MDD is a growing problem in clinical psychiatry (Munos et al. 2009; Safer and Zito 2019; Goh et al. 2020). Another challenge in treating depressed patients is the chronic nature of the disease that often necessitates lifelong drug treatment and, thus, the management of side effects, which may further contribute to the development of certain somatic problems in MDD patients, including type-2 diabetes in TCA-treated patients (Wang et al. 2021) and stroke in SSRI-treated patients (Trajkova et al. 2019), which increase risks of morbidity and death (Insel 2009; Baune et al. 2012; WHO 2012, 2017; Zuzarte et al. 2018).

The predominant pharmacotherapy for MDD remains the enhancement of brain monoaminergic neurotransmission, which is founded on the monoamine hypothesis of depression developed over half a century ago. Whilst the selective antagonism of N-methyl-D-aspartate (NMDA) receptors is a promising new mechanistic framework for treating depressive symptoms, the long drought between scientific breakthroughs for truly novel and effective antidepressant treatments raises important questions regarding the limitations of preclinical studies, and, in particular, the reliability and validity of animal models of depression (Cryan and Slattery 2007; Neumann et al. 2011; Borsini 2012; Wegener et al. 2012; Harro 2013; Harro et al. 2019). Rodents, despite being popular models for preclinical psychiatric research, are ill-suited for a complex understanding of the pathophysiology of human psychiatric diseases (Landgraf 2003; Ellenbroek and Youn 2016). The scarcity of research exploring new target treatments is made worse by a lack of robust or consistent methodologies for generating experimental models. Similarly, the way in which depressive-like behaviors are measured can be widely disparate between research groups (McArthur and Borsini 2006; Demin et al. 2019). Species-specific behavioral features might be due, for example, to the distinct differences in responses of the monoaminergic system, among other mechanisms, to stress in humans and in experimental animals (Barker et al. 1994; Heyman 2007; Harro 2019). Consequently, to succeed in developing novel pharmacotherapies for MDD, it is crucial at the level of preclinical research to identify more robust and translationally valid parameters in animal models of depressive phenotypes.

Chronic mild stress (CMS), an animal model of depression, was first developed in the 1980s (Katz 1981, 1982, 1984; Willner et al. 1987) and has gained favor for several reasons. Firstly, it was founded on the basis of etiological relevance, as the pathology in animals is induced with variable, unpredictable, and uncontrollable chronic stress, an established risk factor for depression (Kessler et al. 2005; Lesch and Mössner 2006). Secondly, the CMS model is associated with the development of anhedonia, a reduction of sensitivity to a reward, which was established as the primary criterion of a depressive-like state in animals (Willner et al. 1987; Willner 1992). Thus, CMS mimics stress-induced behavioral changes that resemble certain key features of MDD, i.e. it displays high face and construct validity. Additionally, CMS-induced depression-like changes can be alleviated with antidepressant treatments, suggesting pharmacological sensitivity of the CMS-induced pathology (Papp et al. 1994, 1996, 2003, 2016; Cryan et al. 2002; McAthur and Borsini 2006; Antoniuk et al. 2019).

The canonic CMS model stems from the original studies by Katz and co-workers who subjected rats to 21 consecutive days of stress-inducing conditions, including electric shocks, immobilization, swimming in cold water, and other strong stimuli, which cause a decrease in sucrose intake, which was interpreted as a sign of a hedonic deficit (Katz 1981). However, there remained a need to develop a model that better simulated the continuous mild stress that is often experienced by humans, and also exhibited anhedonia as a core feature and symptom of depression. To achieve this goal, Willner and his group used a set of milder stressors in which the animals are continually exposed to these micro-stressors in an unpredictable fashion (Willner 2017). The micro-stressors included soiled cage stress, presence of novel objects, group housing, light/dark reversal, noise bursts, restricted access to food, constant lighting, tilted cage, food and water deprivation, scotophoric light, among others. The protocols also extended the stress exposure for up to 3 months (Willner et al. 1987; Willner 1992, 2005). In the original version of the new CMS model, reward sensitivity was evaluated in a free-choice sucrose preference/consumption test following food and water deprivation and was shown to decrease within the first few weeks of exposure. It is of note that the sucrose preference/consumption can be restored to normal levels by treatment with antidepressant drugs (Willner 2016, 2017).

Despite overall extensive use of the CMS model for several decades, the reproducibility of anhedonia and depressive-like syndrome induced with CMS has been frequently reported as insufficient. Even when genetically identical animals are used, studies often report contradictory outcomes and fail to define consistent molecular, neuroanatomical, and physiological phenotypes in either rats or mice (Forbes et al. 1996; Weiss 1997; Reid et al. 1997; Phillips and Bar 1997; Hagan and Hatcher 1997; Holmes 2003; Anisman and Matheson 2005; Strekalova et al. 2005, 2011). In some publications, stressed animals were reported to show “unreliable” decreases in sucrose intake that were are “inconsistent” over time, for instance, in CMS-exposed Wistar and PVG hooded rats (Nielsen et al. 2000). Similar outcomes have been reported in other studies (Matthews et al. 1995; Hatcher et al. 1997; Harris et al. 1997; Harkin et al. 2002; Kompagne et al. 2008). Data on locomotion, anxiety, exploration, and other behaviors in CMS-exposed rodents often demonstrate paradoxical and conflicting behavioral changes, such as anxiolytic-like features in conjunction with decreased scores of helplessness, and discordance between the behavioral phenotype of chronically stressed animals and human symptoms of depression (*for a review, see*: Willner 2005; Anisman and Matheson 2005; Strekalova 2008; Strekalova and Steinbusch 2010; Slattery and Cryan 2017). As a consequence of the issues relating to the reproducibility and inconsistencies of stress-induced anhedonia, as measured by sucrose preference, the implementation and interpretation of the data from CMS models in relation to MDD research has proved problematic (*for a review, see*: Nestler et al. 2002; Cryan et al. 2002; Anisman and Matheson 2005; Borsini 2012; Slattery and Cryan 2014). However, it is clear that these limitations can be overcome if methods are adopted that acknowledge address the issues relating to the variability experienced by researchers.

The aim of this text is to provide a critical review, according to an established framework (Grant and Booth 2009), of CMS studies in rats, including the consideration of rarely reviewed reports on vegetative (somatic) parameters, and to discuss the possible origins of its conceptual and methodological flaws. We also discuss ways of overcoming these limitations by proposing modifications to the model. Utilizing World of Science (WoS) search engine, we conducted a comprehensive search of articles focusing on selected aspects of the CMS paradigm. Briefly, the criteria set for the literature search were based on the number of citations for each work, which was adjusted for publication year to capture more recent studies that are generating interest in the field (*for search details, see further sections*). While this approach can be expected to introduce some biases—for example by the exclusion of important, but poorly cited work—it has enabled the critical appraisal of the mainstream literature that underpins the field and has allowed the limitations of the most commonly employed rat CMS methodologies to be addressed.

### Characteristics of stressors used in the CMS model

The first CMS models were considered particularly problematic because of the inconsistency in the induction of the depressive state in animals. The stressors, which varied in nature, reproducibility and duration are presented from thirty-one extended CMS studies in rats (Table 1). We used WoS search engine to find articles featuring search terms “chronic unpredictable stress”, “chronic mild stress”, and “chronic unpredictable mild stress” to identify these studies. The search criteria applied were as follows: (CUM OR CMS OR CUMS) AND (rat) AND (depression). We limited record types to journal articles only, eliminating reviews and conference proceedings. Only articles with citation numbers of >200 and recent papers (2018-present) with >25 citations that reported the stressors used were studied (*for further search and exclusion criteria see Supplementary Table**S1**, Supplementary Excel File**2*).

**Table 1:** Stressor, behavioral tests, and depressive-like changes reported in CMS studies

Rat Strain	Stress period (weeks)	Stressors				Changes	Author
		Light dark cycle	Social	Housing condition	Physiological		
Sprague-Dawley	4	OI	/	WB (overnight)	Cs (1 hr); Sw (1 hr)l Sc (5 min); Ho (42°C, 5 min); TP (1 min)	FST (↑ immobility time); OFT (↓ time in center); EPM (↓ open arm entries)	Hao et al. 2019
Wistar	9	II (10-14 hrs)	IG (10-14 hrs)	WB (10-14 hrs)	Ct (10-14 hrs); F/W D (10-14 hrs); SI (10-14 hrs)	SCT (↓ sucrose consumption)	Czéh et al. 2018
Wistar	5	OI	/	WB (24 hrs)	Cs (2 hrs); Ct (24 hrs); F/W D (24 hrs); Sc (5 min); Res (2 hrs); ES (0.5 mA, 0.5 sec)	SPT (↓ sucrose preference); FST (↑ immobility time, ↓ swimming time)	Fan et al. 2018
Spargue-Dawley	4	/	CH (24 hrs)	Soc (24 hrs)	Cr/s (1 hr); Ct (24 hrs); Sc (5 min); Co (1 hr); Res (1 hr)	SPT (↓ sucrose preferences); FST (↑ immobility time)	Li et al. 2018
Sprague-Dawley	3	ReLD (24 hrs)	/	/	F/W D (24 hrs); No (1500 Hz, 92 dB, 1 hr); Res (1 hr); TP (1 min)	SPT (↓ sucrose preference); OFT (↓ rearing frequency)	Lu et al. 2018
Sprague-Dawley	4	OI	/	WB (24 hrs)	Ct (7 hrs); F/W D (24 hrs); Sw (5 min); Sc (5 min); Res (2 hr); TP (1 min)	MWM (↑ escape latency)	Shen et al. 2018
Sprague-Dawley	3	OI	/	WB (12 hrs)	Cs (10 min); Ct (12 hrs); F/W D (12 hrs); No (10 min); Res (10 min); TP (10 min)	SPT (↓sucrose preference) ; FST (↑ immobility time); OFT (↓ crossing, grooming and rearing frequency)	Song et al. 2018
Sprague-Dawley	12	ReLD	/	/	Cs (15 min); F/W D (24 hrs); SI (12 hrs); Sc (5 min); Ho (40°C, 5 min); No (radio, 12 hrs); Res (2 hrs);	FST (↑ immobility time); OFT (↓ rearing frequency, ↓ total distance travelled); EPM (↓ time spent in open arms)	Wang et al 2018
Sprague-Dawley	3	LO (3 hrs); OI (ReLD (24 hrs)	CH (24 hrs)	WB (24 hrs)	Ct (24 hrs); F/W D (24 hrs); SI (12 hrs); Sc (5 min); Co (1 hr); Res (4 hrs); TP (1 min)	SPT (↓ sucrose preference); FST (↑ immobility time); OFT (↓ total distance, ↓ central activity, ↓ rearing frequency); EPM (↑ closed arm entries, ↓ open arm entries)	Zhang et al. 2018
Sprague-Dawley	3/7/28 days	LO; OI;	CH; IH	WB	Cr; Ct; FD; WD; SI; Od; Co (1 hr); Swim; Res (1 hr)	SPT (↓ sucrose preference); SCT (↓ sucrose consumption); NOR (↓ nivel object exploration)	Franklin et al. 2018
Sprague-Dawley	5	OI	/	WB (14 hrs)	Ct (3 hrs); F/W D; SI (1 hr); No (radio, 4 hrs)	SPT (↓ sucrose preference); FST (↑ immobility time); SB (↓ social exploration)	Kreisel et al. 2014
Sprague-Dawley	3	LO (3 hrs); OI	CH; IH; IG	/	Cr/s; Ct; Od; F/W D; SI (overnight); Co	SPT (↓ sucrose preference); NSFT (↑ latency to feed)	Li et al. 2011
Sprague-Dawley	5	LO (3 hrs); OI	CH (overnight); IH (overnight)	WB (overnight)	Cr (1 hr); Ct (overnight); Od (overnight);F/W D; SI (overnight); Sc (10 min; (Co (1 hr)	SPT (↓ sucrose preference); AAT (↑ number of escape failures, ↑ latency to escape)	Banasr et al. 2010
Rat Strain	Stress period (weeks)	Stressors				Changes	Author
		Light dark cycle	Social	Housing condition	Physiological		
Wistar	6	OI; ReLD (48 hrs)	/	WB (8 hrs); SoC (1 hr)	Ct (3 hrs); F/W D	SPT (↓ sucrose preference); NSFT (↑latency to feed); FST (↑ immobility time)	Bessa et al. 2009
Sprague-Dawley	5	LO (3 hrs); OI	IH (overnight)	/	Cr (1 hr); Ct (overnight); Od (overnight); FD; WD; SI (overnight); Sc (10 min); Co (1 hr)	SPT (↓ sucrose preference); NSFT (↑ latency to feed); FST (↑ immobility time); AAT (↑ number of escape failures)	Banasr and Duman 2008
Sprague-Dawley	2	/	CH (24 hr); IG (1 hr)/	/	Cs (1 hr); Sw (15 min); Sc (10 min)l Res (30 min); TP (10 min); ES (15 min, 1.5 mA, 15 ecs on, 150 sec off)	EPM (↓ open arms entries); Attentional set-shifting test (↑ trials to reach criteria	Bondi et al. 2008
Sprague-Dawley	3	LO (3 hrs); OI	CH; IH (3 hrs/overnight)	WB	Cr (1 hr); Ct (overnight); Od (3 hr); FD; SI (overnight); Co; Res (1 hr)	SPT (↓ sucrose preference)	Koo and Duman 2008
Sprague-Dawley	8, 15, or 35 days	LO (3 hrs); OI	CH (overnight); IH (overnight)	WB (overnight(	Cr (1 hr); Ct (overnight); Od (overnight);F/W D; SI (overnight)l Sc (10 min); Co (1 hr)	SPT (↓ sucrose preference); ↑ plasma corticosterone	Banasr et al. 2007
Wistar	10	ReLD; II (8-12 hrs)	CH (8-12 hrs)	SoC (8-12 hrs)	Ct (8-12 hrs); F/W D (8-12 hrs); SI (8-12 hrs); No (white, 8-12 hrs)	SCT (↓ sucrose consumption)	Bortolato et al. 2007
Sprague-Dawley; Wistar	24 days	II (10-14 hrs)	IG (10-14 hrs)	WB (10-14 hrs)	Ct (10-14 hrs); F/W D (10-14 hrs); SI (10-14 hrs)	SCT (↓ sucrose consumption)	Lucas et al. 2007
Sprague-Dawley	2	/	CH (24 hrs); IH (24 hrs)	/	Cs (1 hr); Sw (15 min); Sc (10 min); Res (30 min); TP (10 min); ES (10-15 min, 1.5 mA, 15 ecs on, 150 sec off)	SPT (↓ sucrose preference)	Lu et al. 2006
Wistar	4	II (10-14 hrs)	IG (10-14 hrs)	WB (10-14 hrs)	Ct (10-14 hrs); F/W D (10-14 hrs); SI (10-14 hrs)	SCT (↓ sucrose consumption)	Jayatissa et al. 2006
Wistar	2	/	CH (overnight); IH (overnight)	/	Cs (1 hr); Sw (15 min); Sc (5 min); Co (1 hr); Res (30 min); Hy (30 min)	↓ weight gain; ↑ adrenal cortical responses to ACTH	Ulrich-Lai et al. 2006
Long Evans	3	/	IH (18 hrs)	/	Cr (3 hrs); F/W D (18 hrs); SI (30 min); Forced swim (5 min); No (white, 30 min); Res (30 min)	MWM (↑ escape latency, ↓ reveral learning)	Hill et al. 2005
Wistar	6	II (10-14 hrs)	IG (10-14 hrs)	WB (10-14 hrs)	Ct (10-14 hrs); F/W D (10-14 hrs); SI (10-14 hrs)	SCT (↓ sucrose consumption); FST (no change in immobility time); OFT (↓ moving duration, ↓ rearing duration); ↓ weight gain	Dalla et al. 2005
Rat Strain	Stress period (weeks)	Stressors				Changes	Author
		Light dark cycle	Social	Housing condition	Physiological		
Sprague-Dawley; Wistar	7	OI	CH (overnight)	WB (overnight); FO (overnight)	Ct (5 hrs); Od (overnight);F/W D; SI (2-5 hrs); Co (30 min); No (white, 3 hrs)	SPT (↓ sucrose preference); SCT (↓ sucrose consumption); ↓ weight gain	Bekris et al. 2005
Wistar	3	/	CH (overnight); IH (overnight)	/	Cs (1 hr); Sw (30 min); Sc (5 min); Co (1 hr); Res (1 hr)	↓ weight gain; ↑ plasma corticosterone	Heine et al. 2004
Wistar	9	II (10-14 hrs)	IG (10-14 hrs)	WB (10-14 hrs)	Ct (10-14 hrs); F/W D (10-14 hrs); SI (10-14 hrs)	SCT (↓ sucrose consumption)	Papp et al. 2003
Lister Hooded	9	OI	IG (24 hrs)	WB (17 hrs)	Ct (7 or 8 hrs); F/W D (19 hrs); FD (23 hrs); WD (7 or 20 hrs); Res (18 or 21 hrs)	SCT (↓ sucrose consumption); EPM (no change in open/closed arm entries); SB (↓ aggression, ↑ submission to the intruder, ↓ social interaction)	Daquila et al. 1994
Wistar	7 or 8	II (10-14 hrs)	IG (10-14 hrs)	WB (10-14 hrs)	Ct (10-14 hrs); F/W D (10-14 hrs); SI (10-14 hrs)	SCT (↓ sucrose consumption)	Papp and Moryl 1994
Lister Hooded	4	OI	CH	WB (17 hrs)	Cr (7/17 hrs); F/W D; No (white, 85 dB, 3 hrs); SI (7/9/17 hrs)	SCT (↓ sucrose consumption); PCT (↓ preference for reward-associated place)	Papp et al. 1991

/ = none applied

Thirty-one studies utilizing CMS procedures on male rats reported depressive-like behavioral changes in sucrose test and/or forced swim test, or depressive-like physiological changes such as increased plasma corticosterone level. Recent studies (published since 2018 and with greater than or equal to 25 citations) are indicated by bold font. This table summarizes a variety of employed stress protocols and a battery of behavioral tests. Stressors used are grouped into 4 categories: Light-dark cycle: LO, lights off; OI, overnight illumination; ReLD, reverse light-dark cycle; II, intermittent illumination; Social: CH, crowded housing; IH, isolation housing; IG, intruder grouping; Housing condition: WB, wet bedding; CS, confined space; SoC, soiled cage; FO, foreign object; Physiological: Cr/s, cage rotation or cage shaking; Ct, cage tilt; Od, aversive odor; F/W D, food and/or water deprivation; FD, food deprivation; WD, water deprivation; SI, stroboscopic illumination; Sw, warm swim; Sc, cold swim; Co, cold room; Ho, hot room; No, noise; Res, restraint; Hy, hypoxia; TP, tail pinch; ES, electrical shock. /- None applied. Length of stressor exposure is indicated in brackets where information is available. Employed behavioral tests and reported depressive-like changes: SPT, sucrose preference test; SCT, sucrose consumption test; NSFT, novelty-suppressed feeding test; FST, forced swim test; PCT, place conditioning test; SB, social behaviors; OFT, open field test; MWM, Morris water maze; EPM, elevated plus maze; AAT, active avoidance test; NORT, novel object recognition test; ↑ an increase, ↓ a decrease of a parameter

In these studies, twenty-seven different stressors were chronically applied in timeframes ranging from 3 days to 10 weeks. In each CMS protocol, between 5 and 14 different stressors were administered to the same cohort. These stressors were applied in an unpredictable fashion, or in some experiments, as a sequence that was repeated weekly. Most of these CMS protocols were based on the original CMS procedure and would not be compliant with the need to achieve consistency/reproducibility between centers or even between consecutive studies in the induction and evaluation of anhedonia (Willner 1992, 2005, 2016, 2017).

Fine tuning of the experimental setup is required to accommodate broad inter-strain and inter-batch variability of animals to various stressors, as well as by variable seasonal and environmental factors (Raab et al. 1986; Nielsen et al. 2000; Kelliher et al. 2000; Alter et al. 2008; Cao et al. 2010; Dalla et al. 2011; Duclot et al. 2011; Franceschelli et al. 2014; Akimoto et al. 2019; Antoniuk et al. 2019; Rao and Androulakis 2020; Armario et al. 1995). For example Armario and co-authors (1995), in five inbred strains of rat, has described important inter-strain differences in the forced swimming behaviour and endocrine responses that could be expected to alter the outcome of CMS studies. Moderation of the duration and nature of the stressors in CMS protocols to alter the severity of the stress have been helpful in the regulation of stress load and can help to overcome some of the intrinsic variability associated with certain inbred and outbred strains (Table 1; *for a review, see:* Gambarana et al. 2001; Strekalova et al. 2011; Harro 2019; Demin et al. 2019). CMS protocol modifications include isolation stress (Domeney and Feldon 1998; Coudereau et al. 1999; Weiss et al. 2000; Von Frijtag et al. 2000), restraint (Klenerova et al. 2003; Qui et al. 2004; Pawluski et al. 2012), social defeat (Meerlo et al. 1999; Von Frijtag et al. 2000; Krishnan et al. 2007; Cline et al. 2015; Riga et al. 2015), exposure to ultrasonic sounds with negative emotional valence (Morozova et al. 2016; Pavlov et al. 2019; Costa-Nunes et al. 2020), administration of LPS (Couch et al. 2016), and other manipulations (Table 1).

Reduced body weight in the CMS-exposed group can be viewed as a possible marker of sufficient stress load that can lead to the induction of anhedonia in rodents (Vitale et al. 2009; Luo et al. 2008; Strekalova and Steinbusch 2010; Hu et al. 2017). Decreased hedonic responsiveness following CMS is generally not considered to be secondary to loss of body weight (*for a review see*: Strekalova et al. 2011; Willner and Belzung 2015; Antoniuk et al. 2019). This is also supported by the findings of similar body weight changes in cohorts of animals resilient and susceptible to stress-induced anhedonia (Bergstroem et al. 2007; Bisgaard et al. 2007; Jayatissa et al. 2008, 2009, 2010; Akimoto et al. 2019). While decreased body weight cannot be considered as a criterion for induction of a depressive state, many researchers find this feature accompanying depressive-like behavior in CMS paradigms, prompting its use as a criterion for adjusting stress intensity in the CMS protocols. Recent stepwise discriminant analysis of the CMS study on Sprague–Dawley rats strongly supports this view (Hu et al. 2017). Other reports suggest no body weight changes in CMS-exposed rodents displaying anhedonia (*for a review, see* Willner 1992). These controversial findings may derive either from the use of dissimilar methods of assessing hedonic deficit, or other methodological shortcomings (Mattews et al. 1995; Forbes et al. 1996; Nielsen et al. 2000). Independent studies are still needed to address such discrepancies.

It is thought that stress procedures of insufficient intensity and duration may not evoke a depressive-like state, but rather lead to other behavioral alterations, for example increased signs of anxiety and general hyperactivity (*for a review, see:* Anisman and Matheson 2005; Strekalova and Steinbush 2009; Slattery et al. 2012). Therefore, when certain facets of depression are observed, behavioral effects reported in experiments with CMS should be interpreted with caution (*for a review, see:* Cabib 1997; Holmes 2003; Slattery et al. 2007, 2012).

The choice of stressors used to produce CMS has been extensively discussed in the literature. The unpredictable and uncontrollable features of the chronic stress paradigm were chosen to simulate the mixed nature of the stressors that can contribute to depression, and, as such, the method seeks to fulfill the construct validity requirements of an animal model of depression (*for a review, see:* Cryan et al. 2002; McArthur and Borsini 2006; Willner 2016). With depression models in rodents, stress uncontrollability and unpredictability are the two impediments for the ability of an animals to adapt to stressors (*for a review, see:* Anisman and Matheson 2005; Heyman 2007). Therefore, we propose the following basic principles for proper stressor selection: (i) the use of ethologically relevant stressors with an emotional component (predation stress, social stressors, ultrasound stress of negative emotional valence), rather than predominantly physical/physiological stressors (restraint stress, foot shock, food and water deprivation); (ii) the omission of stressors that are likely to confound outcomes in behavioral tests (e.g. omission of food and water deprivation; thermal stressors that are known to affect the accuracy of the sucrose test; foot shock stress and repeated swimming are inappropriate when memory and Porsolt swim tests, respectively, are performed); and (iii) the omission of poorly tunable stressors, such as continuous lighting, wet bedding, or social defeat. Several chronic stress models, which adhere to the principles recommended above, have been developed and are worth considering in any experiment planning, e.g. new variants of social stress (Finnell et al. 2018; Nakatake et al. 2020), maternal separation in a combination with other stressors (Rüedi-Bettschen et al. 2006; Diamantopoulou et al. 2018; Houwing et al. 2019), variants of social isolation (Berry et al. 2012; Yang et al. 2016; Stevenson et al. 2019), as well as the ultrasound stress paradigm (Zorkina et al. 2019; Demaestri et al. 2019; Pavlov et al. 2019).

To summarize, the reliability and reproducibility of CMS, as a rodent model of MDD, can be improved by selecting stressors that are more controllable in terms of duration and severity. However, the etiological relevance and methodological compatibility with the behavioral tests use to establish the depressive-like state need to be considered. This approach, however, may often conflict with local animal welfare regulations that explicitly impose restrictions on the spectrum of CMS modifications available for implementation and thus we recommend that investigators build the need for adjustments into their protocols for consideration at ethical review. The failure to induce a hedonic deficit in a cohort of animals is likely to increase overall usage and harm and thus a typical example and a worst case example should always be presented in an application to conduct CMS experiments.

### Sucrose test and possible confounds in behavioral assessment of anhedonia

Several approaches have been adopted to evaluate the ability of a rodent to experience pleasure, including progressive ratio responding, intracranial self-stimulation, conditioned place preference, and intake of palatable food (*for a review, see:* Cryan et al. 2002; Hu et al. 2017; Belovicova et al. 2017; Harro 2013, 2019; Antoniuk et al. 2019). All these techniques have been used in CMS studies to assess rodent sensitivity to a reward. However, in the present work we excluded them from discussion here because they are rarely used, or exhibit extreme variability in the methodology, or because limited number of reports are available for analysis (*for a review, see:* Barnes et al. 2014). The sucrose preference paradigm is most frequently employed as a method for assessing anhedonia in the CMS models, as rodents have been demonstrated to drink sweetened water avidly. This is a mechanistically undemanding experimental test based on a two-bottle, free-choice paradigm, in which reduced sucrose intake and preference are taken as signs of anhedonia (*for a review, see:* Willner 1992, 2016, 2017; Antoniuk et al. 2019). Unlike other the other methods mentioned, this approach addresses hedonic sensitivity, rather than reward-seeking behavior, and does not depend on learning, anxiety, and locomotion that are frequently altered in the CMS-exposed animals.

Initially, the sucrose test was developed as a sensitive assay to measure reward sensitivity in mice (Levine 1967; Stockton and Whitney 1974; Harriman 1976) and then used in rat preclinical paradigms, including the CMS model (Katz 1981; Willner et al. 1987; Krimm et al. 1987; Pucilowski et al. 1993). Compared to mice, rats have been reported to display lower variability in the sucrose test (*for a review, see:* Strekalova and Steinbusch 2009, 2010; Scheggi et al. 2018). However, the sucrose test protocols employed in the literature vary greatly in test duration, sucrose concentration, and mean data in control groups (Table 2).

**Table 2:** Measured parameters of sucrose test reported in CMS studies

**Rat strain**	**Stress period (weeks)**	**Sucrose concentration (%)**	**Adaptation to sucrose (days)**	**Food and water deprivation prior test (hrs)**	**Test duration (hrs)**	**Mean of sucrose intake (g)**		**Mean of sucrose preference (%)**			**Author**
						**Stress group**	**No stress control**	**Stress group**	**Baseline**	**No** **stress control**	
**Wistar**	**9**	**1.5**	**35**	**14**	**N.A.**	**5**	**14**	**N.A.**	**N.A.**	**N.A.**	Czéh et al. 2018
**Wistar**	**5**	**1**	**2**	**24**	**3**	**N.A.**	**N.A.**	**50**	**N.A.**	**90**	Fan et al. 2018
**Sprague- Dawley**	**4**	**1**	**2**	**4**	**1**	**N.A.**	**N.A.**	**55**	**N.A.**	**80**	Li et al. 2018
**Sprague- Dawley**	**3**	**1**	**4**	**24**	**1**	**N.A.**	**N.A.**	**40**	**N.A.**	**82**	Lu et al. 2018
**Sprague- Dawley**	**3**	**1**	**N.A.**	**12**	**12**	**N.A.**	**N.A.**	**70**	**N.A.**	**100**	Song et al. 2018
**Sprague- Dawley**	**3**	**1**	**N.A.**	**N.A.**	**24**	**N.A.**	**N.A.**	**55**	**80**	**80**	Zhang et al. 2018
**Sprague- Dawley**	**3/7/28 days**	**1**	**2**	**4**	**1**	**N.A.**	**N.A.**	**66**	**N.A.**	**84**	Franklin et al. 2018
Sprague-Dawley	5	2	N.A.	N.A.	3	N.A.	N.A.	67	N.A.	76	Kreisel et al. 2014
Sprague-Dawley	3	1	2	4	1	N.A.	N.A.	50	N.A.	65	Li et al. 2011
Sprague-Dawley	35 days	1	2	4	1	N.A.	N.A.	69	N.A.	75	Banasr et al. 2010
Wistar	6	1	7	18	1	N.A.	N.A.	75	90	90	Bessa et al. 2009
Sprague-Dawley	35 days	1	2	4	1	N.A.	N.A.	33	N.A.	75	Banasr and Duman 2008
Sprague-Dawley	3	1	2	4	1	N.A.	N.A.	35	N.A.	70	Koo and Duman. 2008
Sprague-Dawley	8, 15, or 35 days	1	2	4	1	N.A.	N.A.	33	N.A.	75	Banasr et al. 2007
**Rat strain**	**Stress period (weeks)**	**Sucrose concentration (%)**	**Adaptation to sucrose (days)**	**Food and water deprivation prior test (hrs)**	**Test duration (hrs)**	**Mean of sucrose intake (g)**		**Mean of sucrose preference (%)**			**Author**
						**Stress**	**No stress group control**	**Stress group**	**Baseline**	**No** **stress control**	
Wistar	10	1	N.A.	15	1	7	12	N.A.	N.A.	N.A.	Bortolato et al. 2007
Sprague-Dawley; Wistar	24 days	1.5	35	18	1	7	15	N.A.	N.A.	N.A.	Lucas et al. 2007
Sprague-Dawley	2	1	N.A.	2.5	1	N.A.	N.A.	60	N.A.	80	Lu et al. 2006
Wistar	4	1.5	35	18	1	7.5	16	N.A.	N.A.	N.A.	Jayatissa et al. 2006
Wistar	6	1	28	14	1	5	10	N.A.	N.A.	N.A.	Dalla et al. 2005
Wistar	7	1	7	23	1	2	5	45	66	66	Bekris et al. 2005
Sprague-Dawley	7	1	7	23	1	2.8	3.2	40	78	75	
Wistar	9	1	10 1-hr sessions	14	1	7.3	14	N.A.	N.A.	N.A.	Papp et al. 2003
Lister Hooded	9	1	2	19	1	6	10	N.A.	N.A.	N.A.	Daquila et al. 1994
Wistar	7 or 8	1	5 1-hr sessions	14	1	7	13.5	N.A.	N.A.	N.A.	Papp and Moryl, 1994
Lister Hooded	4	0.7	N.A.	20	1	6	8	N.A.	N.A.	N.A.	Papp et al. 1991

Reviewed twenty-five studies utilizing CMS procedures on male rats employed various modifications of 1-h sucrose consumption test, where concentrations of sucrose solution, duration of water deprivation period and measured parameters were variables. The results demonstrated a broad range of changes across compared studies in stressed and non-stressed groups

Modifications of 1-h sucrose preference test protocols were reported in twenty-five studies utilizing CMS procedures on male rats. Observed variety of experimental modifications regarding concentration of sucrose solution, duration of a deprivation period and other parameters might result in broad range of values in stress vs. non-stressed groups across studies. Abbreviation: N.A. - Information is not available. Recent studies (published since 2018 and with greater than or equal to 25 citations) are indicated by bold font

Of the 31 papers meeting the search criteria (*see previous section*) that used the sucrose test, twenty-five of the CMS studies employed different methodologies and the test outcomes (Table 2). Studies often employed water deprivation ranged from 4 to 24 hrs, prior to testing, and twelve of the studies used periods of longer than 20 hrs, which might be considered a serious design flaw (Jensen et al. 2013). For examples of the variability reported in these tests, Banasr et al. (2007, 2008) and Koo and Duman (2008) (Wang, 2008 #55) reported 33–35% sucrose preference in stressed animals and 70-75% sucrose preference in control animals, whereas Song et al. (2018), Banasr et al. (2010), and Bessa et al. (2009) demonstrated 69–75% sucrose preference in stressed animals and 75-100% sucrose preference in control animals. It is of note that a drop in sucrose preference below the chance level (50%) in two-bottle paradigms is likely to mirror the avoidance response of rodents towards sucrose rather than lowered reward sensitivity, and, thus, is likely to be indicative of potential artifacts in the experimental design. Based on our evaluation of these studies, a standardized experimental protocol and the criteria for measuring anhedonia in the rat CMS model that are adjusted for the strain used and for the operating requirements of a specific lab would be useful. This has also been highlighted in recent reviews following a survey of the users of the CMS model (Willner 2016, 2017).

Variabilities in sucrose test results in rodents can be explained by substantial inter-strain and inter-batch differences in experimental animals, as well as the high sensitivity of drinking behavior and sucrose intake to internal and external conditions (*for a review see:* Koprdova et al. 2016; Antoniuk et al. 2019). Factors that may impact on the outcome of these test include: (**i**) effects of stressors present during the sucrose test or the lasting action of previously applied stressors on consummatory behavior (Shaham et al. 1993; Kant and Baumann 1993; Schoenecker et al. 2000); (ii) sugar concentration (Stockton and Whitney 1974; Harriman 1976); (iii) diet and water deprivation (Muscat and Willner 1992; Hatcher et al. 1997; Jensen et al. 2013), (iv) neophobia (Krimm et al. 1987; Strekalova 2021); (v) social status of rodents (Raab et al. 1986; Strekalova et al. 2004; Tonnissar et al. 2006); (vi) sensitization to reward experiences during repeated or prolonged exposure to palatable solutions (Harriman 1976; Strekalova and Steinbusch 2009, 2010; Strekalova 2021); (vii) fluctuations in calorie and water intake due to differences in body mass and altered equilibrium of sympathetic/parasympathetic regulation (Tonissaar et al. 2006); (viii) strain and inter-individual variabilities in liquid and sucrose intake (Kant and Baumann 1993; Coudereau et al. 1999; Nielsen 2000; Brennan et al. 2001; Glendinning and Gresack 2002; Tonissaar et al. 2006; Pucilowski et al. 1993); (ix) circadian rhythms (Stephan and Zucker 1972; Kant and Baumann 1993; Daquila et al. 1994; Strekalova and Steinbusch 2010; Strekalova 2021); and (x) inter-batch variability (Nielsen et al. 2000; Jakovcevski et al. 2008; Robinson 2009; Theilmann et al. 2016; Kõiv et al. 2019).

In a number of CMS protocols, food and water deprivation prior to the sucrose preference test was shown to suppress sucrose intake and preference in naïve rats (Matthews et al. 1995; Forbes et al. 1996; Harris et al. 1997). Furthermore, a limiting point within the sucrose test is the natural inter-individual variability in circadian patterns of liquid intake. Notably, as in individual laboratory rodents, liquid intake peaks during different parts of the day (Kant and Baumann 1993; Strekalova et al. 2011, 2021). The evaluation of sucrose solution intake during a 1-3 hrs-long sucrose test may, therefore, introduce a systematic error into the results. Altered lighting conditions may affect not only rodent circadian rhythmicity, but could serve as an additional source of variability in the sucrose consumption behavior (Jensen et al. 2013). Additionally, reiterative sucrose tests, either weekly (e.g. over 4–6 weeks) or in prolonged sessions of 24–72 h, as well as the use of sucrose concentrations over 2%, were found to induce a ‘ceiling effect’ on sucrose solution intake, which can greatly affect test sensitivity (Strekalova et al. 2006; Slattery and Cryan 2017).

Other critical limiting aspects of the sucrose test in rodents are physical factors, such as bottle leakage, which can substantially affect results and may occur due to high home cage activity induced by CMS. Uncontrolled fluctuations in room temperature can generate significant shifts in innate drinking behavior and bottle leakage. For instance, when tap water with an average temperature of 17Cﹾ is used as solvent for sucrose, the difference between the water temperature and the laboratory room temperature (22Cﹾ in average) peaks at 5Cﹾ. This margin is sufficient to cause a gradual increase of air pressure in the bottle as the solutions warm up during the test, pushing liquid out of the bottle and resulting in significant error in drinking behavior evaluation (Strekalova 2008; Strekalova and Steinbusch 2009, 2010). In addition, poorly controlled air temperature can affect humidity and the drinking behavior of rats. This factor is even more marked when stress and control groups are housed in separate rooms with slightly different temperatures, which should not occur in the experimental design. Another important parameter that is often neglected in sucrose test methodology is control over scents that contaminate sugar. For example, storing sugar in the vicinity of chemicals or in a plastic bag can lead to absorption of undesirable flavors and cause taste aversion in animals, leading to reduced sucrose intake and preference (Strekalova 2008).

While the reduction of a preference for and intake of highly palatable substances is sensitive to antidepressants, an analysis of the literature suggests that there is considerable variability of these measures in rodents exposed to CMS (for a review see: Holmes 2003; Belovicova et al. 2017; Scheggi et al. 2018; Antoniuk et al. 2019). Indeed, in a review by Willner it was noted that ‘it is certainly the case that sucrose intake is more variable, and therefore less accurate, in mice than in rats’ (2017) and some evidence-based guidelines do exist for increasing the accuracy of the test in rat studies, which suggests that animals ought to be screened before CMS to exclude those (typically around 20%) with very low, very high, or very variable sucrose intake (Papp 2012).

Despite sucrose preference being an established tool to stratify hedonic behavior, a recent qualitative and quantitative analysis of 153 studies that used the CMS paradigm and sucrose preference test has demonstrated a large heterogeneity in the responses of individual animals (Antoniuk et al. 2019). While this analysis has not revealed the differences in validity of sucrose intake and preference in manifesting anhedonia in the CMS-exposed rodents, one should consider that under-reported negative results may compromise the analysis of the literature. At the same time, recent studies showed that, similar to mice, rats display individual patterns of liquid intake at different times of the day (Tonissaar et al. 2006), which, in the Wistar strain, was also shown to be present in both genders (Strekalova 2021). Moreover, sucrose intake in laboratory rats can be confounded by the novelty factor, and equally, by repetitive access to the sucrose solution (Strekalova 2021). Therefore, while methodological studies directly comparing the accuracy of the two parameters have not been published, it can be hypothesized that the use of sucrose preference as a measure, instead of sucrose intake, might help reduce a variability in the outcome from the sucrose test. The highest reliability of this test in rats can be achieved by selecting optimal test parameters, such as concentration of sucrose solution, frequency/duration of the test, and time of testing with respect to the light cycle (Papp 2012; Willner 2017). Mathematical analysis reveals that there is greater variability in sucrose intake compared to sucrose preference (see Supplementary file), which is supported by studies in rats and mice (Strekalova and Steinbusch 2009, 2010; Strekalova 2021). Although rats showed lower circadian variability in sucrose preference than in sucrose intake, the latter parameter was reported to be less variable at an individual level (Tonissaar et al. 2006).

Finally, it is worthwhile to pointing out that in some studies, e.g. Henningsen et al. (2013), there have been unexpected increases in sucrose consumption and preference in the stressed rats. Similar findings have been reported in other studies at least at some points in the induction process of anhedonia (Matthews et al. 1995; Strekalova et al. 2004, 2006). These effects are interpreted as manifestation of stress-induced diabetes mellitus (Schoenecker et al. 2000) and hyper-compensatory ‘pro-hedonic’ response to stress (Willner 2005, 2016). They might also be associated with hypersecretion of corticotropin-releasing factor and vasopressin in hypothalamus and hypophysis (Cole and Koob 1994; Gizowski et al. 2016), which provokes behavioral invigoration, stronger consumption response, and stress-induced thirst (Strekalova et al. 2004, 2006). Thus, for these reasons, it is suggested that employing the sucrose preference parameter in addition to sucrose intake measure may, potentially, reduce possible distortions in the evaluation of the hedonic status of CMS rats.

Here, we have presented specific features of drinking behavior in rats, and some flaws in experimental design that can confound sucrose preference test results. Based on the issues discussed, the artifacts in the sucrose test in rats may be alleviated by: (i) the evaluation of the hedonic state in the CMS model based on sucrose preference as the primary measure as previously suggested (Weiss 1997; Ferreira et al. 2018); (ii) refinement of the test duration to within the limits of 5–12 h, instead of 1–2 h or 28–48 h; (iii) restriction of repetition of the test sessions on the same animal and elongation of inter-test time windows to avoid the “ceiling effect”; (iv) the use of sucrose solutions with concentrations of 1% or less (Papp 2012); (v) synchronizing the testing time with the dark (active) phase of an animal’s light cycle (Tonissaar et al. 2006); (vi) elimination of potential physical artifacts by ensuring similar temperatures of the drinking solutions and of air in the holding rooms (e.g. by filling the drinking bottles in advance and keeping them in the same room where the testing takes place for a few hours, storing sucrose and washing bottles in scent-controlled conditions (Strekalova and Steinbusch 2010), and (vii) mitigation of effects of the preceding stressors, such as food and water deprivation 12–24 h before the test.

### Other behavioral endpoints and challenges in measuring CMS-induced behaviors in rats

The majority of reports using the CMS paradigm in rats demonstrate ‘classic’ depressive-like changes beyond anhedonic behavior that mimic other human symptoms of depression, such as helplessness and psychomotor inhibition (Fig. 1). Table 1 summarizes the information on the changes in these behaviors, including those evaluated in Porsolt forced swim test and the open field test.

The forced swim test has been widely employed as an assessment of ‘despair’ and helplessness in small rodents, which, with some limitations (*for a review see*: Gambarana et al. 2001, Cryan et al. 2005; Bogdanova et al. 2013; Yankelevitch-Yahav et al. 2015; de Kloet and Molendijk 2016; Demin et al. 2019; Ferreira et al. 2018), manifest as a prolongation of passive floating behavior (immobility) and reduced latency to stop swimming and start floating (Cryan et al. 2005).

We used WoS search engine to find articles featuring search terms “chronic unpredictable stress”, “chronic mild stress”, “chronic unpredictable mild stress”, and “forced swim test.” The search criteria applied were as follows: (CUM OR CMS OR CUMS) AND (rat) AND (depression) AND (FST). We limited record types to journal articles only, eliminating reviews and conference proceedings. Only articles with citation numbers of >80 were studied. We also included articles from the first section of this review (Table 1) that reported FST as a measure of CMS-induced depressive phenotype in rats (*for further search and exclusion criteria see Supplementary Table**S2**, Supplementary Excell File**2*). In order to avoid any misleading sematic cues in the literature search, we used the commonly accepted term “despair behavior” to indicate “immobility” /”floating behavior” (Unal and Canbeyli 2019). However, we accept that the use of ‘despair behavior’ might be considered by some to be problematic because of its anthropomorphic connotations and we also accept that a degree of caution is warranted when using this term (Commons et al. 2017; Molendijk and de Kloet 2019).

Table 3 summarizes the behavioral parameters, including duration of floating and floating latency, reported in twenty-one CMS experiments. Notably, this comparison reveals substantial variability in the reported means of the duration of floating behavior: from 29 s (Song et al. 2018) to 480 s (Chad et al. 2010) in stressed groups and from 5 s (Song et al. 2018) to 160 s (Silva et al. 2008) in control animals. The use of antidepressants was shown to reduce the duration of floating in CMS groups (Harro and Kiive 2011). The latency of floating was suggested to enhance the sensitivity of detecting depressive-like changes in rodents (Castagne et al. 2009; Porsolt et al. 2001; Strekalova et al. 2016; Markova et al. 2017; Ferreira et al. 2018). The measurement of helplessness in the forced swim test can be affected by various factors (for a review see: Bogdanova et al. 2013; Yankelevitch-Yahav et al. 2015), e.g. stress-induced hyperactivity (Igarashi and Takesha 1995; Hata et al. 1999; Kelliher et al. 2000, Strekalova et al. 2005; Schweizer et al. 2009), and a non-specific increase in impulsive behavior and locomotion that can be exposed in chronically stressed rodents by behavioral analysis (*see below*). Future CMS research would benefit from methodological studies addressing possible rodent physiology-driven errors in the measurement of immobility behavior in CMS rats.

**Table 3:** Measured parameters of FST reported in CMS studies

**Rat strain**	**Stress period (weeks)**	**Immobility time (sec)**		**Latency to immobility (sec)**		**Swimming time (sec)**		**Climbing time (sec)**		**Duration of Pre-test (min)**	**Duration of test (min)**	**Author**
		**Stress group**	**No stress control**	**Stress group**	**No stress control**	**Stress group**	**No stress control**	**Stress group**	**No stress control**			
**Sprague-Dawley**	**4**	**161**	**119**	**/**	**/**	**85**	**120**	**56**	**63**	**15**	**5**	**Hao et al.** 2019
**Wistar**	**5**	**230**	**100**	**/**	**/**	**40**	**100**	**/**	**/**	**15**	**5**	Fan et al. 2018
**Sprague-Dawley**	**4**	**105**	**20**	**/**	**/**	**/**	**/**	**/**	**/**	**15**	**5**	Li et al. 2018
**Sprague-Dawley**	**3**	**29**	**5**	**/**	**/**	**/**	**/**	**/**	**/**	**15**	**6**	Song et al. 2018
**Sprague-Dawley**	**12**	**220**	**130**	**/**	**/**	**55**	**150**	**/**	**/**	**/**	**5**	Wang et al. 2018
**Sprague-Dawley**	**3**	**125**	**100**	**/**	**/**	**/**	**/**	**/**	**/**	**15**	**5**	Zhang et al. 2018
Sprague-Dawley	3	120	20	/	/	175	100	/	/	15	5	Yue et al. 2017
Sprague-Dawley	5	170	45	/	/	/	/	/	/	15	5	Liu et al. 2015
Sprague-Dawley	5	165	135	50	70	/	/	/	/	/	5	Kreisel et al. 2014
Sprague-Dawley	4	140	65	/	/	/	/	/	/	15	5	Chang and Grace 2014
Sprague-Dawley	3	180	/	/	/	30	/	/	/	/	10	Greene et al. 2009*
Wistar	8	204	72	/	/	/	/	/	/	15	5	Karson et al. 2013
Sprague-Dawley	3	175	115	/	/	85	100	40	85	15	5	Garza et al. 2012**
Sprague-Dawley	3	236	92	/	/	/	/	/	/	15	5	Bai et al. 2012
**Rat strain**	**Stress period (weeks)**	**Immobility time (sec)**		**Latency to immobility (sec)**		**Swimming time (sec)**		**Climbing time (sec)**		**Duration of Pre-test (min)**	**Duration of test (min)**	**Author**
		**Stress**	**No group stress control**	**Stress group**	**No stress control**	**Stress group**	**No stress control**	**Stress group**	**No stress control**			
Sprague-Dawley	4	400	/	/	/	390	/	90	/	/	15	Larsen et al. 2010*
Sprague-Dawley	4	480	/	/	/	/	/	/	/	/	15	Chad et al. 2010*
Wistar	6	125	75	/	/	/	/	/	/	10	5	Bessa et al. 2009
Wistar	6	125	75	/	/	/	/	/	/	10	5	Bessa et al. 2009
Sprague-Dawley	5	160	100	/	/	/	/	/	/	15	5	Banasr and Duman 2008
Wistar	3	110.5	51.6	/	/	/	/	27.9	98.0	15	5	Yang et al. 2016
Sprague-Dawley	3	60	25	/	/	/	/	/	/	/	5	Luo et al. 2008
Wistar	2	250	160	30	70	/	/	/	/	10	5	Silva et al. 2008
Sprague-Dawley	3	115	70	/	/	/	/	/	/	/	5	
Wistar	6	75	85	/	/	170	170	/	/	15	5	Dalla et al. 2005

Analysis of twenty-four studies with CMS model revealed a diversity of protocols and a variety of measured read-outs in the employed forced swim test, as well as variable outcome in both stressed and non-stressed groups of rats. Recent studies (published since 2018 and with greater than or equal to 25 citations) are indicated by bold font./ - Not applicable.* - did not include a no-stress control group, ** - used mean counts (dominant behavior of each 5-s interval)

The second most frequently used assay for examining CMS-induced behavioral changes in rats is the open field test. Even though the majority of studies report reduction in both vertical and horizontal movements in CMS rats (Table 1), which suggests the presence of psychomotor inhibition as observed in MDD, these and other locomotor changes in laboratory rodents can be observed in stressed rats with no signs of hedonic deficit (*for a review see:* Willner 2005; Slattary and Cryan 2014; Belovicova et al. 2017; Harro 2019) and therefore cannot be directly attributed to clinically relevant depressive-like changes. Some studies report unaltered open field behavior or increases in locomotion and grooming activity (Igarashi and Takesha 1995), suggesting behavioral invigoration of CMS-exposed rats that contradicts the concept of psychomotor inhibition as a classic feature of a depressive-like state (Ellenbroek and Youn 2016). It has to be pointed out that either positive or negative alterations in general locomotion of CMS-exposed rodents are not readily extrapolated to human-specific symptoms of depression and often do not correlate with major molecular and key behavioral features of this disease (Schweizer et al. 2009; Strekalova et al. 2011; Hu et al. 2017). As discussed above, signs of anhedonia, as measured by different methods, constitute a major facet of depression as proposed by Hamilton back in 1967. However, the abundant literature (erroneously) refers to the changes in the open field behavior—either decreases or increases—as ‘depression-like’ changes following original publications of Katz (1981, 1982). As such, for the sake of improving validity and methodology of evaluating open field activities in CMS-exposed rats, further experimental refinement of this model is needed. This is particularly important in the context of the reported hyperactivity induced by chronic stress in rats (Spasojevic et al. 2016; Zhan et al. 2019; *see also below*).

While increased anxiety is considered a comorbid condition rather than a clinical sign of depression, the assessment of a CMS-induced behavioral phenotype typically employs a battery of tests that concomitantly seek to measure anxiety-like changes (Table 1). Besides central activity in the open field, anxiety-like changes in CMS-exposed rats have also been measured in the elevated plus/O-maze, dark/light box, novelty suppressed feeding/drinking test, and others. The majority of studies demonstrate signs of elevated anxiety in stressed rats, while some report lack of such changes or ‘anomalous’ pro-anxiolytic effects of CMS, as reviewed in earlier work by Willner (2005). Most anxiety tests are locomotion-based, and the open field is a good example to demonstrate that test-specific behavior is influenced by both anxiety and locomotion (Ohl et al. 2001; Landgraf 2003; Neumann et al. 2011). In many studies, however, no effort was made to discern the two parameters, thus complicating interpretation of the results. However, there is clearly value in assessing anxiety and locomotion activity in the open field in conjunction with the Porsolt forced swim test to associate or dissociate the behaviors in these two tests to gain further insight into what motivational, cognitive, and emotional deficits are present.

Apart from evaluating anhedonia, helplessness, locomotor activity, and anxiety, CMS studies have utilized numerous models to study other behavioral changes that are characteristic of depressive-like state, as in learning and memory, as well as social and sexual features and consumption behavior. These assays reveal both decreases and increases in learning scores, intake of water and diet, and changes in escape behavior (*for a review, see*: Soblosky and Thurmond 1986; Willner 2005, 2016, 2017; Gambarana et al. 2001; Hu et al. 2017; Belovicova et al. 2017). Thus, the CMS experience, typically, has a profound effect on a number of behavioural characteristics, though there is considerable variation between reports.

While the majority of reports report that CMS induces ‘classic’ depressive features in the behavior of rats, a substantial number of studies reveal inconsistencies in development of these changes (*for a review, see*: Weiss and Simson 1986; Cabib 1997; Harris et al. 1997; Nestler et al. 2002; Anisman and Matheson 2005; Slattery and Cryan 2014; Harro 2019; Demin et al. 2019). This phenomenon could be partly due to the limited accuracy of behavioral tests, inter-individual variability of stress response, and the development of general hyperlocomotion that were previously described in murine CMS models (Strekalova et al. 2005; Schweizer et al. 2009; Boulle et al. 2014), which are discussed further below.

### Hyperlocomotion as a source of artefacts in the CMS paradigm

In many studies that have employed chronic stress to induce depression there are reports of atypical changes in behavior (*for a review see*: Willner 2005; Strekalova and Steinbusch 2009; Ferreira et al. 2018), which appear to contradict well-established behavioral profiles of locomotor inhibition, signs of despair, and elevated anxiety-like changes, classical symptoms of depression in humans (Kessler and Bromet 2013; Pacchiarotti et al. 2020) and stress in rodents (Ohl et al. 2001; Neumann et al. 2011). For instance, Wistar rats exposed to CMS were shown to linger in anxiety-related areas of the elevated plus maze and the dark/light box, suggesting incongruous ‘anxiolytic-like’ changes (Spasojevic et al. 2016; Zhan et al. 2019). Similarly, CMS-exposed rats were reported to show ‘anomalous’ increase in struggling and decrease of floating in the Porsolt forced swim test (Koprdova et al. 2016; Wainwright et al. 2016). Importantly, both anti-depressant drugs and psychomotor stimulants are known to alter immobility behavior of stressed animals in the Porsolt forced swimming test, suggesting the invocation of a generalised invigoration and hyperactivity induced under these conditions (*for a review see*: Cryan et al. 2005; Bogdanova et al. 2013).

In addition to the ‘anomalous’ behavioral effects of CMS in mice (Strekalova et al. 2005), it has also been reported that rats exposed to CMS can display non-specific hyperlocomotion and general behavioral invigoration as a response to the slightly stressful procedure of behavioral testing (Igarashi and Takesha 1995; Chaouloff et al. 1995; Bertoglio and Carobrez 2002). Bright light, cold water, novelty, handling, and other factors were found to trigger hyperactivity in chronically stressed, but not in naïve small rodents, often confound all behavioral measurements (Willner 2005; Strekalova and Steinbusch 2010; Ferreira et al. 2018). Indeed, systematic studies with variable illumination conditions revealed differential locomotory, exploratory, and anxiety-related behavioral changes in male Wister rats studied under lighting intensities that ranged from 44 lux to 600 lux (Bertoglio and Carobrez 2002) and in Sprague-Dawley rats, which were exposed to lighting with intensity ranging from 75 lux to 500 lux (Weiss et al. 2000). The role of lighting in rat floating/swimming behavior was demonstrated in the forced swim test; moreover, excessive escape-oriented behavior in CMS rats in this test was found to be reversible by antidepressants (Kelliher et al. 2000). In a translational context, this phenomenon has been considered as a manifestation of agitated depression, a clinical form of depressive disorder (Pacchiarotti et al. 2020; Sampogna et al. 2020). Other testing conditions during behavioral analyses can also trigger hyperlocomotion in CMS rodents. For example, Igarashi and Takesha (1995) showed that not only light brightness, but also handling experience can prevent excessive ambulation and defecations of CMS-exposed rats in the open field test.

Strain and sex differences in locomotor responses of CMS-exposed rats can, additionally, account for inconsistencies in classical features of stress-induced anxiety-like and despair behaviors, as suggested by a number of studies (Chaouloff et al. 1995; Weiss et al. 2000; Dalla et al. 2011; Franceschelli et al. 2014; Martis et al. 2018) and reviewed in recent literature (Willner et al. 2016, 2017; Harro 2019; Antoniuk et al. 2019). It has been proposed that a specific type of stress can play a role in behavioral invigoration effects; for instance, isolation stress in Wistar rats was shown to induce locomotion after encountering unknown objects in Sprague-Dawley rats (Domeney and Feldon 1998; Weiss et al. 2000).

Collectively, having some control over the impact of the testing environment on rodent stress can, potentially, be of great help in increasing the reliability and reproducibility of behavioral studies of CMS-exposed rats. Environmental stress accompanying behavioral analyses of stressed rats can be reduced by switching to ‘mild’ testing conditions, such as: low illumination intensity of behavioral apparatuses, ambient temperature, and low depths of water in the swimming tank in the Porsolt test; larger sizes of swimming tanks in this test; frequent handling; limited height of testing elevated apparatuses used for the assessment of anxiety-like behavior; exposure to novelty; limited number of tests applied in each animal per day; and other factors. As for mice (Strekalova et al. 2011), the additional tuning of behavioral protocols for rats to reduce extraneous stress may aid in avoiding serious artifacts in the analysis of CMS-induced features.

### Vegetative (somatic) effects of CMS

Vegetative (somatic) symptoms and associated changes in sleep and metabolic regulation are of great clinical importance, as they can be predictive of either characteristic or abnormal psychiatric trajectories, including worsening life quality and shortening life span (for a review see: Airaksinen 1999; Baglioni et al. 2011; Baune et al. 2012; Pequignot et al. 2016; Dell’Osso et al. 2014). Remarkably, the CMS paradigm was shown to successfully mimic many of these aspects of depression in rats, which is of paramount importance for understanding their physiology, validation of CMS models, and exploration of new therapies (*for a review see*: Weiss and Simson 1986; Moreau 2002; Grippo 2009; Strekalova et al. 2009). Table 4 summarizes the analysis of the literature addressing MDD-like changes in rat CMS studies.

**Table 4:** CMS-induced vegetative (somatic) effects

Somatic functions investigated	Rat strain	Stress period (weeks)	Stressors				Depressive-like changes	Corroborating behavioural tests	Author
			Light-dark cycle	Social	Housing condition	Physiological			
EEG	Wistar	3	RELD (24 hrs)	/	/	Ct (7 hrs); FD (24 hrs); WD (24 hrs); Cw (5 min); TP (1 min); ES (1 mA, 10 sec/shock, 10 times w/t 10 sec intervals)	Thalamal and cortical EEG (↓ power at theta and delta bands, ↓ thalamus → cortex information flow)	SCT, MWM	Quan et al. 2011
Heart Functions	Sprague-Dawley	2/4	/	/	WB (18 0r 24 hrs); New cage (18 or 24 hrs)	Ct (18 or 24 hrs); FD (18 or 24 hrs); WD (18 or 24 hrs); Sw (10 or 15 min); Res (2 or 3 hrs)	↑ body temperature; ↓ heart rate variability; ↑ sympathovagal balance (LF/HF); ↑ QT interval	SPT	Park et al. 2017
	Wistar	8	II (10-14 hrs)	CH (10-14 hrs)	SoC (10-14 hrs)	Ct (10-14 hrs); F/W D 910-14 hrs); SI (10-14 hrs)	↑ heart rate; ↔ blood pressure	SCT, OFT	Matchkov et al. 2015
	Sprague-Dawley	4	OI	CH (16 or 20 hrs)	WB (16 hrs)	Ct (7 hrs); WD (17 hrs); SI (4 or 6 hrs); No (white, 3 or 4 hrs);	↑ heart rate; ↓heart rate variability; ↔ baroreceptor reflex control of heart rate; ↓ lumbar sympathetic nerve activity in response to baroreceptor unloading	SPT	Grippo et al. 2012
	Sprague-Dawley	4	/	IG	/	Ct (6 hrs); Od; FW (24 hrs); SI (5 hrs)	↔ heart rate; ↑ mean arterial blood pressure; ↑ pressor and tachycardic responses to air jet stress	/	Cudnoch-Jedrzejewska et al. 2010
	Sprague-Dawley	4	OI	CH (7 or 48 hrs)	WB (12 hrs)	Ct (7 hrs); WD (18 hrs); SI (4 or 6 hrs); No (white, 5 hrs);	↑ heart rate; ↓ heart rate variability; ↑ vulnerability to ventricular arrhythmias	SPT	Grippo et al. 2003
	Sprague-Dawley	4	OI	CH (20 or 70 hrs)	WB (17 hrs)	Ct (4 hrs); FD (20 hrs); WD (20 hrs); SI (2 or 3 hrs); No (white, 3 hrs)	↓ heart rate variability; ↑ bradycardia following β-adrenergic receptor blockade; ↑ tachycardia following cholinergic receptor blockade; ↑ pressor and tachycardic responses to stress	SPT	Grippo et al. 2002
Thermo-regulation	Wistar	3.5	II (10-14 hrs)	CH (10-14 hrs)	WB (10-14 hrs)	Ct (10-14 hrs); F/W D (10-14 hrs); SI (10-14 hrs)	↓ nocturnal core body temperature	SCT	Christiansen et al. 2016
	Sprague-Dawley	4	LO (36 hrs); OI (36 hrs)	/	WB (19 hrs)	Ct (15 and 5 hrs); FD (24, 17, 8 and 4 hrs); WD (17, 8 and 4 hrs);	Diminished circadian fluctuation in rectal temperature	SCT, SPT	Ushijima et al. 2006
Sleep	Wistar	8 days	Unspecified (10 5-min episodes)	/	/	Ct (every 12 hrs); No (85 dB, radio)	↓ slow-wave sleep duration; ↔ REM sleep duration	/	Guesdon et al. 2006
	Sprague-Dawley	4	OI (36 hrs)	IG (2 hrs)	WB (21 hrs); New cage (21 hrs)	Ct (3 hrs); FD (4 hrs or overnight); WD 4 hrs or overnight)	↑ REM sleep, waking and slow-wave sleep duration; ↑ REM and waking episodes; ↔ latency to enter REM sleep	SCT	Gronli et al. 2004
	Lister Hooded	5	OI (18 hrs)	/	CS (18 hrs); SoC (18 hrs)	Ct (6 or 24 hrs); F/W D (18 hrs); WD (6 hrs); SI (16.5 hrs)	↓latency to enter REM sleep; ↑ REM sleep; ↑ sleep stage fragmentation	/	Cheeta et al. 1997
	Wistar	33 days	OI	CH (overnight)	SoC (overnight)	F/W D (overnight; WD (overnight)	↓latency to enter REM sleep; ↑ REM sleep; ↑ number of REM episodes; ↔ delta power of slow-wave sleep	Ventral tegmentum self-stimulation	Moreau et al. 1995

Analysis of thirteen CMS studies addressing vegetative (somatic)depressive-like changes in rats demonstrates a diversity of stress protocols used and variable outcomes in terms of the occurrence of changes and their nature. Majority of studies with CMS demonstrate that this model recapitulated the key features of vegetative (somatic) changes associated with MDD (see the text). Stressors acronymed as in the Table 1. /- Not applied. Abbreviations: *REM sleep* rapid eye movement sleep, *SWS* slow wave sleep

To extracted CMS studies that employ vegetative function measures, the following query term was applied as an additional filter to the search conducted for Table 1: "AB= (heart rate) OR (electrocardiogram) OR (ECG) OR (blood pressure) OR (core body temperature) OR (rectal temperature) OR (thermoregulat*) OR (circadian rhythm*) OR (sleep) OR (sympathetic) OR (parasympathetic))". The extracted studies were then manually screened to generate Table 4. In the case where multiple studies published by the same laboratory utilizing the same CMS procedure and vegetative function parameters were returned by the search, only the most comprehensive studies were included in the table. As it is not the purpose of the current review to provide a comprehensive summary of the effects of CMS procedure on each of the vegetative functions in rats, this table is not an exhaustive summary of all studies conducted to date. Only the most relevant studies were included (*for further search and exclusion criteria see, Supplementary Excell File**2*).

One of the well-established somatic effects of exposure to CMS is the shortening of latency and elongation to the rapid eye movement (REM) sleep phase. This is a well-known feature that can precede actual depressive episodes, and can persist during remission (Baglioni et al. 2011). Decreased slow-wave sleep duration, diminished sleep continuity, and multiple changes in sleep architecture are also characteristic for MDD (Wang et al. 2015a, b; Zhang et al. 2017). These features have been recapitulated in a number of CMS rat models. For example, decreased latency of REM was shown in Wistar rats, which was reversed by the administration of antidepressants (Cheeta et al. 1997). Gronli et al. (2004, 2012) reported an increase in REM duration and sleep fragmentation, shortened slow-wave sleep duration, and an increased number of wake episodes, all of which have been corroborated by other studies in CMS-rodents (Guesdon et al. 2006; Cline et al. 2015).

With reference to sleep dysregulation, depression is characterized by aberrant circadian rhythmicity (Nechita et al. 2015). It was found that CMS procedures can mimic circadian rhythm disturbances that occur during depression as manifested by altered patterns of melatonin and corticosterone secretion, rectal temperature rhythmicity, and general activity (Gorka et al. 1996; Avery et al. 1999, Ushijima et al. 2006; Couch et al. 2013). The most common findings are a flattening of normal circadian fluctuations of core body temperature, secretion of glucocorticoids and melatonin (Ushijima et al. 2006; Pechlivanova et al. 2010; Christiansen et al. 2016), which are strongly associated with depression in clinical reports (Branchey et al. 1982; Chen et al. 2018; Høifødt et al. 2019). Circadian rhythmicity aberrations often coincide with the onset of depressive syndrome in humans (Klenerova et al. 2003; Kolasa et al. 2014; Jia et al. 2019) and are closely related to a dysregulated balance of sympathetic/ parasympathetic tone (Baune et al. 2012).

Elevated sympathetic regulation during depression is a well-studied clinical feature that is considered to be the principal cause of the high comorbidity observed between depression and cardiovascular diseases (Baune et al. 2012; Péquignot et al. 2016). Elevated sympathetic tone results in tachycardia, arrhythmia, elevated sensitivity of arteries to catecholamines, and altered baroreflex response. These changes were recapitulated in rat CMS studies, which also revealed prolongation of local field potential duration in both cardiac tissue and thoracic T1–5 spinal cord nerves, pathologic changes in the myocardium (Hu et al. 2011; Liu et al. 2018), prolonged Q-to-T wave intervals (Park et al. 2017; Liu et al. 2018) and other changes (Bouzinova et al. 2012; Frey et al. 2014). Of note, the aforementioned changes can be reversed by the administration of antidepressant drugs (Crestani et al. 2011).

The attenuation of baroreflex was shown during depression and is known to result in increased risk of ventricular fibrillation (Billman et al. 1982; Airaksinen 1999), possibly owing to a reduction in parasympathetic activity and simultaneous increase in sympathetic activity in patients with MDD (Watkins and Grossman 1999; Pitzalis et al. 2001). CMS studies have reported reduced baroreflex sensitivity, elevated blood pressure, and increased arterial sensitivity to noradrenaline in stressed rats, further supporting validity of this model in mimicking somatic consequences of human depression (*for a review see*: Grippo et al. 2009).

Considered together, these findings substantiate the validity of the CMS model as the changes to the autonomic nervous system mimic those seen in humans with MDD. Given the need for increasing consistency in the reporting of CMS-induced behavioral changes, and the technical advancements in monitoring equipment that have become less costly, the use of objective measures of vegetative (somatic) functions and sleep ought to be considered as an attractive outcome measure in any CMS study. Studies addressing such aspects of CMS may also benefit from the stratification of animals that are ‘resilient’ or ‘susceptible’ to the CMS procedure, since stress without depressive-like changes can affect a variety of assessed parameters. Taking into account the intriguing data on sex differences following CMS on vegetative regulation (Baker et al. 2006; Franceschelli et al. 2014; Santangeli et al. 2016), exploring gender-related effects could also reveal more useful data to guide future research.

### Stratification of CMS animals into ‘resilient’ and ‘susceptible’ phenotypes

Numerous studies, which have social defeat stress, predation stress, chronic social instability stress, administration of glucocorticoids, have reported marked interindividual variability in the response of an animal to stress that is suggestive of the presence of a susceptible AND resilient phenotype (Strekalova 1995; Taliaz et al. 2011; Steimer and Driscoll 2005; Jackovevsky et al. 2008; Duclot et al. 2011; Theilmann et al. 2016; Scherholz et al. 2020; Rao and Androulakis 2020; Labaka et al. 2021). For example, in rats, chronic exposure to social defeat immediately elevates intracranial self-stimulation, which can be used to assess of reward threshold as a measure of anhedonia. The thresholds are found to remain elevated in a subset of susceptible rats, but the thresholds in resilient rats are only acutely elevated during the initial period of social defeat are then, subsequently, unaffected despite ongoing stress exposure. Thus stratification according to susceptible vs. resilient is found to be useful in other paradigms (Der-Avakian et al. 2014).

As it was recently outlined by Willner, some variability in the CMS model could be considered a strength as long as the results are reproducible (Willner et al. 2016, 2017). Indeed, clinical practice points to a large variability in the vulnerability and resistance to mood disorders including depression among individuals with a clinical history of stress (Lesch and Mössner 2006; Feder et al. 2009). Within the CMS model, categorizing animals as individually ‘susceptible’ or ‘resilient’ to the development of stress-induced depressive-like state was first proposed for mice (Strekalova et al. 2004) and subsequently has found application in other stress models (Krishnan et al. 2007; Schmidt et al. 2010), including the CMS model in rats (Jayatissa et al. 2008; Tonissaar et al. 2006; Delgado et al. 2011; Taliaz et al. 2011; Herrera-Pérez et al. 2012; Sun et al. 2017; Raya et al. 2018; Tang et al. 2019; Zurawek et al. 2017, 2019). As the development of depressive-like syndrome is recorded in 50-70% of stressed animals, the ‘resilient’ group can serve as internal control for the isolated effects of stress (Strekalova et al. 2004, 2011).

This new approach to CMS design, in comparison to the original model, has helped resolve an obvious conceptual drawback of this depression paradigm, where all changes found in chronically stressed group are naturally attributed to the depressive-like (anhedonic) state. Yet, distress *per se* does not correspond to depressive state, but instead can be associated with a number of physiological alterations, which are not necessarily related to depressive syndrome (Bergström et al. 2007; Bisgaard et al. 2007; Jayatissa et al. 2006, 2008, 2009; Henningsen et al. 2009; Sterlemann et al. 2010; Cao et al. 2010; Delgado et al. 2011; Kolasa et al. 2014; Wang et al. 2015a, b; Palmfeldt et al. 2016; Zurawek et al. 2017, 2019; Martis et al. 2018; Tang et al. 2019). As such, while the original CMS paradigm was subject to this flaw, the proposed stratification of CMS animals based on their vulnerability to stress, theoretically, transforms the issue into an informative feature.

This refined modification of the CMS model is clearly advantageous to addressing the mechanisms of the resilience to stress-induced depression, which, in resilient individuals, might involve circuits and pathways of the stress response that are distinct from those of susceptible individuals (Delgado et al. 2011; Strekalova et al. 2011; Palmfeldt et al. 2016). Studies of the last decade using a comparison of ‘resilient’ versus ‘susceptible’ cohorts of rats elucidated a large portion of neurobiological basis for these distinct profiles of response to the CMS. Among numerous findings, individual vulnerability to stress-induced anhedonic state in rats was found to correlate specifically with aberrant expression of SERT-related miRNA regulatory mechanisms in the mesocortical circuit (Zurawek et al. 2017), compromised brain expression of somatostatin and prolactin receptors (Faron-Górecka et al. 2016), elevated secretion of CRH and Urocortin 2 (Kolasa et al. 2014), altered hippocampal expression of 5-HT1A receptor and its epigenetic regulation (Zurawek et al. 2019; Gorinski et al. 2019), and glucocorticoid and cannabinoid receptors (McLaughlin et al. 2013; Sun et al. 2017).

Other features ‘resilient’ versus ‘susceptible’ cohorts include changes in response to psychostimulants, dopamine agonists, brain expression of dopamine D2 receptor, turnover and binding ability of beta-adrenergic receptor (Willner 2005; Cao et al. 2010), deviant neuroanatomical features and interactions between the hippocampus and prefrontal cortex (Delgado et al. 2011; Bessa et al. 2013; Kafetzopoulos et al. 2018). There are also general proteomic changes in the hippocampal region (Bisgaard et al. 2007), including alterations in mitochondrial and metabolic processes (Tang et al. 2019), reduced brain expression of BDNF, vascular endothelial factor, and other neuroplasticity markers (Bergström et al. 2007; Jayatissa et al. 2008, 2009, 2010; Taliaz et al. 2011; Sun et al. 2017), as well as increased expression of immediate early genes in the medial prefrontal cortex (Palmsfeldt et al. 2016). Together, these data suggest that subgroups of individuals resistant to induction of depressive phenotype in experimental paradigms of depression can be employed as an internal control to improve simulation of depressive states in animal models. Additionally, differentiating between resilient and susceptible animals of inbred laboratory lines allows for exploring epigenetic and post-translational mechanisms of stress resilience, which was not feasible with the original CMS protocol by R. Katz and P. Willner. Studies employing this method have revealed new important biomarkers of depression and potential therapeutic targets that can aid in the development of personalized therapeutic regimes (Mill and Petronis 2007; Alter et al. 2008; Feder et al. 2009; Demin et al. 2019).

Remarkably, while the issue of the inter-individual variability in response to CMS is now well-established in the literature, and that the advantages of taking this inherent variability into account within the experimental design is well documented, our literature analysis suggests that most researchers are reluctant to adopt measures that make allowances for this variability (Tables 1, 2, 3, 4). This may contribute to the inconsistent validity and reproducibility that has been reported in the field of CMS depression studies. Moreover, a search in WoS using the basic criteria applied for Table 1, with 2 additional search terms “susceptible” and “resilient”, resulted in 17 publications, and, from this list, less than a half employed behavioral methods beyond the sucrose test (Table 5). The categorizing criteria and the percentage of rats classified as either susceptible or resilient varied greatly across these studies; in the most cases, post-CMS changes in sucrose consumption, relative to a baseline level, were used as criteria to stratify the groups. All the studies reported marked differences between CMS-susceptible vs. resilient cohorts in terms of the changes in their sucrose drinking behaviour before and after CMS exposure. A diversity of molecular parameters was investigated; four studies adopted high-throughput metabolomics or proteomics approaches and reported distinctive profiles among susceptible, resilient and control cohorts (Akimoto et al. 2019; Henningsen et al. 2012; Zhang et al. 2021; Palmfeldt et al. 2016). Other studies focused on more specific mechanisms including HPA axis activity (Christiansen et al. 2012), GABAergic neurotransmission (Czéh et al. 2018; Nieto-Gonzalez et al. 2015), c-Fos activation (Febbraro et al. 2017), neurogenesis (Jayatissa et al. 2009), and microRNA expression (Zurawek et al. 2017). Most studies compared control, susceptible and resilient groups in all pairwise combinations. 10 studies reported molecular features that distinguish the susceptible group from the resilient and control groups (Christiansen et al. 2012; Czéh et al. 2018; Delgado y Palacios et al. 2014; Febbraro et al. 2017; Li et al. 2014; Nieto-Gonzalez et al. 2015; Remus et al. 2015; Sun et al. 2017; Yu et al. 2020; Zurawek et al. 2017). 3 studies also identified molecular signatures that distinguish the resilient group from the susceptible and control groups (Czéh et al. 2018; Febbraro et al. 2017; Zurawek et al. 2017). These stratification studies, although scarce in number, provide evidence that stress susceptibility and resilience are likely to be underpinned by distinctive molecular mechanisms. Thus, further studies are required to explore the molecular basis of susceptibility and resilience, which would further help to justify the use of stratification principles in CMS studies.

**Table 5:** Stratification of CMS-rats to ‘resilient’ and ‘susceptible’ phenotypes upon anhedonic features in the sucrose test

**Rat strain**	**Stress period (weeks)**	**Stratification criteria and percentage of CMS subgroups**		**Behavioural differences between susceptible and resilient subgroups**	**Other differences between susceptible and resilient subgroups**	**Author**
		**Susceptible**	**Resilient**			
Wistar/ST	4	<65% sucrose preference on day 29 (50%)	<10% change in sucrose preference from baseline (44.4%)	Susceptible rats displayed ↓ rearing in OFT than resilient rats but the difference was not statitically significant. Both susceptible and resilient rats displayed ↓ number of line crossings and ↑ grooming time in OFT than control rats.	↓ Weight gain in both susceptible and resilient rats compared to control rats. Susceptible and resilient rats differed from control rats and from each other in the hippocampal metabolite profiles. 12 metabolites were measured, among which N-acetylaspartate ↑ in the hippocampus in both resilient and susceptible groups compared to the control group, and the differences were more prominent in the susceptible group. Aspartate, acetate and GABA ↓ in the hippocampus in both resilient and susceptible groups. No change in hippocampal BDNF level in any CMS subgroups compared to control group.	Akimoto et al. 2019
Wistar	8	CMS rats were categorized based on their averaged sucrose index (avSI, averaged ratio between weekly sucrose intake and baseline sucrose consumption) into 3 subgroups: resilient (avSI=1.01 ± 0.06, 20%), intermediate (avSI=0.71 ± 0.03, 55%), and susceptible (avSI=0.56 ± 0.03, 25%).		None conducted.	All CMS rats were heterogeneous in their diural corticosterone secretion rhythm regardless of subgroups. Susceptible rats had higher corticosterone secretion and less efficient HPA axis negative feedback than resilient rats during the course of CMS but the difference dimished by the end of CMS procedure.	Christiansen et al. 2012
Wistar	9	>30% ↓ in sucrose comsumption from baseline	No decrease (or even sometimes increase) in sucrose consumption from baseline.	Susceptible rats failed to learn in the object-place paired-associate task over 30 days indicated by no change in the maximum number of consecutive correct trials, whereas control rats displayed gradual improvement. Resilient rats were not tested.	Susceptible rats had ↓ medial prefrontal cortex (mPFC) GABAergic input, GABA release, GABAB receptor mediated inhibition than resilient and control rats. Susceptible rats had ↓ number of palvalbumin positive cells in the infralimbic cortex than resilient and control rats. Susceptible rats had ↓ number of cholecystokinin positive cells in the cingulate gyrus than resilient and control rats. Resilient rats had ↑ mumber of neuropeptide Y positive cells in all mPFC subregions than susceptible and control rats. Both susceptible and resilient rats had ↓ number of calretinin positive cells in the IL than control rats.	Czéh et al. 2018
Wistar	8	Significant ↓ in sucrose consumption compared with control and resilient rats	No difference in sucrose consumption compared with control rats	None conducted.	Susceptible rats had ↓ diffusion kurtosis and ↑ axial diffusion in the caudate putamen and ↑ radial diffusion in the amygdala than resilient and control rats. Susceptible rats had higher caudate putamen-to-whole brain volume ratio than resilient and control rats.	Delgado y Palacios et al. 2014
Wistar	4	>30% ↓ in sucrose comsumption from baseline	<10% ↓ in sucrose comsumption from baseline	None conducted.	Susceptible rats had ↑ expression of c-Fos in the amygdala, medial habenula, and IL than resilient and control rats. Resilient rats had ↓ expression of c-Fos in lateral and ventral orbital cortices than susceptible and control rats. Both susceptible and resilient rats had ↓ expression of c-Fos in magnocellular ventral lateral geniculate nucleus.	Febbraro et al. 2017
Wistar	8	>30% ↓ in sucrose comsumption from baseline (43%)	<10% ↓ in sucrose comsumption from baseline (23%)	None conducted.	Susceptible rats and resilient rats exhibited distinct hippocampal proteomic profiles.	Henningsen et al. 2012
Wistar	4 or 8	>40% ↓ in sucrose comsumption from baseline	No change in sucrose consumption from baseline.	None conducted.	↓ Total cell number and ↓ BrdU+ cells in the granual cell layer of ventral hippocampus was comparable in resilient rats and susceptible rats.	Jayatissa et al. 2009
Sprague– Dawley	3	>25% ↓ in sucrose comsumption from baseline (78.8%)	<10% ↓ in sucrose comsumption from baseline (21.2%)	Susceptible rats had ↓ travel distance and ↓ traveling speed in OFT than resilient and control rats.	Susceptible rats had ↓ weight gain than resilient and control rats. Susceptible rats had ↓ hippocampal EphA4 protein and ↑ ephrinA3 protein levels than resilient and control rats.	Li et al. 2014
Long Evans	9	>30% ↓ in sucrose comsumption from baseline (41%)	<10% ↓ in sucrose comsumption from baseline (20%)	Susceptible rats displayed impaired task acquisition in Different Paired- Associates Learning task compared to resilient and control rats.Resilient rats displayed ↑ impulsivity-like behaviours in Different Paired-Associates Learning task compared to susceptible and control rats.	No other significant difference reported.	Martis et al. 2018
Wistar	8	>40% ↓ in sucrose comsumption from baseline (50%)	<10% ↓ in sucrose comsumption from baseline (50%)	None conducted.	Susceptible rats had ↓ GABA release probability and spontaneous GABAergic activity in hippocampal granule cells than resilient and control rats. No change in the number of parvalbumin-positive interneurons or the kinetics of miniature inhibitory postsynaptic currents in any CMS subgroups compared to control group.	Nieto-Gonzalez et al. 2015
Wistar	8	>30% ↓ in sucrose comsumption from baseline (55%)	<10% ↓ in sucrose comsumption from baseline (24%)	None conducted.	Susceptible rats and resilient rats exhibited distinct PFC synaptosome proteomic profiles. Susceptible rats had ↓ PFC synaptosome GFAP protein expression than resilient and control rats.	Palmfeldt et al. 2016
Fischer- 344	10 days	Significant ↓ in sucrose consumption compared with control rats (34.8%)	No difference in sucrose consumption compared with control rats (65.2%)	None conducted.	Post-CMS overnight food and water deprivation ↓ sucrose preference and sucrose intake in both resilient and susceptible rats, with the effects being more prominent in susceptibles rats. Post-CMS overnight food and water deprivation ↑ IL-1β protein levels in the hippocampus of both resilient and susceptible rats, and in the hypothalamus of susceptible rats only. The adrenal weight and plasma epinepherine level of both resilient and susceptible rats similarly ↑ compared to control rats, while the plasma corticosterone level was not significantly changed.	Remus et al. 2015
Sprague– Dawley	8	>30% ↓ in sucrose comsumption from baseline (33%)	<10% ↓ in sucrose comsumption from baseline (20%)	Susceptible rats spent ↓ time in the center and performed ↓ line crossings in OFT than resilient and control rats.	Susceptible rats had ↑ hippocampal mGluR5 mRNA and protein levels, and ↑ hippocampal glucocorticoid receptor protein level than resilient and control rats.	Sun et al. 2017
Wistar	3	>25% ↓ in sucrose comsumption from baseline (53.8%)	<10% ↓ in sucrose comsumption from baseline (46.2%)	None conducted.	↓ Weight gain in both susceptible and resilient rats compared to control rats. No change in mPFC or hippocampal BDNF protein levels in either subgroup compared to control rats.	Theilmann et al. 2020
Sprague– Dawley	10/20/30 days	Rats with the lowest 30% of the sucrose preference of all CMS rats after 20 days of stress.	Rats with the highest 30% of the sucrose preference of all CMS rats after 20 days of stress.	Susceptible rats displayed ↑ immobility time in FST, and ↑ latency to feed in NSFT than resilient and control rats.	Susceptible rats had ↓ density of perineuronal net in the prelimbic cortex than resilient and control rats.	Yu et al. 2020
Wistar	8	>30% ↓ in sucrose comsumption from baseline	No significant difference in sucrose consumption from baseline.	Susceptible rats displayed ↑ immobility time in FST than resilient and control rats. Susceptible rats displayed ↓ number of rearing and ↓ number of line crossings in OFT than resilient and control rats.	276 proteins were found to be differentially expressed between resilient, susceptible and control groups. Bioinformatics analysis revealed that the biological processes of these differential proteins were related to mitochondrion organization, protein localization, coenzyme metabolic process, cerebral cortex tangential migration, vesicle- mediated transport.	Zhang et al. 2021
Wistar Han	2	>20% ↓ in sucrose comsumption from baseline (70%)	No decrease (or even sometimes increase) in sucrose consumption from baseline. (30%)	None conducted.	Expression levels of miR-18a-5p, miR-34a-5p, miR-135a-5p, miR-195-5p, miR-320-3p, miR-674-3p, and miR-872-5p ↑ in the VTA, and ↓ in the mPFC in all CMS rats compared to control rats. Resilient rats had higher VTA expression of miR-195-5p, miR-320-3p and miR872-5p, and lower mPFC expression of miR-320-3p and miR872-5p than susceptible rats. ↓ SERT protein in VTA in all CMS rats and more pronounced in resilient rats compared to susceptible rats.	Zurawek et al. 2017

Seventeen studies utilizing CMS procedures on male rats described a stratification of stressed animals to "susceptible" and "resilient" upon signs of anhedonia in the sucrose test (with an exception of one study that additionally defined an “intermediate” group). This Table summarizes diverse criteria of susceptibility / resilience to anhedonic behaviour in various strains of rats exposed to CMS of variable duration. The criteria of stratification and the percentage of animals assigned to the subgroups of “susceptible” or “resilient” individual rats greatly vary across the studies. All studies have reported marketable differences between CMS-"susceptible" and "resilient" cohorts in depressive-like features, even more often changes in both subgroups are distinct from non-stressed control rats. Remarkably, only seven out of seventeen publications, have addressed behavioural parameters

Overall, further attempts to develop modifications of the CMS paradigm by adjusting the stratification principle to the ongoing research would be beneficial. It is becoming increasingly evident that categorizing laboratory animals as ‘resilient’ and ‘susceptible’ promises more accurate and organized identification of new targets and biomarkers for pharmacotherapy of depression, and more effective assessment of new antidepressant treatments.

## Conclusions and future perspectives

There is a growing body of evidence that the CMS paradigm can be generally regarded as a valid animal model of a depressive-like phenotype, and that chronic stress methods offer advantages in translational studies of depression pathophysiology and research for new antidepressant therapies. We believe that, based on our review of the available literature, a necessity exists to refine the methods of applying stress and evaluating behavior. Foremost among these amendments should be the stratification of animals into ‘resilient’ and ‘susceptible’ with regards to depressive-like changes induced by the CMS protocol. This approach is commonly applied in gene and protein expression profiling studies, but not in those of basic model parameters, e.g. of vegetative (somatic) features of CMS. Meanwhile, recent studies showed the role of vegetative symptoms in suicidality among depression patients, suggesting the importance of studying this underexplored aspect of depression. Additionally, this approach expands the horizons of pre-clinical studies aimed at differentiating between the therapeutic effects of antidepressants for depression symptoms and other concomitant neuropsychiatric changes, e.g. elevated anxiety. Variations of sensitivity to antidepressant treatments in ‘resilient’ and ‘susceptible’ stressed rats can greatly aid in pharmacological characterization and differentiation of new drug candidates in future research.

It is important to emphasize the fact that the behavioral assessment of face, construct, and predictive validity of CMS is reliable when the sucrose test for assessing anhedonia is performed accurately. In a previous review (Antoniuk et al. 2019), the authors have summarized the basic principles for ensuring better sucrose test precision, and maintaining reliability of this key test within the frame of CMS studies.

The present analysis of the literature suggests that there is plenty of scope for improving the reliability and reproducibility of the CMS model in rats by employing appropriate experimental testing conditions. As for mice, CMS can cause occasionally reported 'anomalous' behavioral profiles in rats, distorting the manifestation of classical depressive-like features of helplessness, elevated anxiety, and locomotor inhibition. As such, more systematic studies focusing on the nature of above-discussed general invigoration effects of CMS on rat behavior might prove fruitful in the development of more accurate protocols of behavioral testing of helplessness, anxiety, memory, and general activity in CMS-exposed rats.

Generally, as any single animal model using small rodents is of limited value in simulating mental disorders, only the implementation of several principally distinct paradigms can improve our insight into the neurobiology of MDD by elucidating the research object from different angles. In this context, the CMS model, which mimics a key depressive feature, anhedonia, is indispensable in pre-clinical depression research. The CMS paradigm, despite its limitations, has been successfully used in drug development and a constellation of interdisciplinary research to obtain insight into the neurobiology of depression. Its potential to increase our understanding of the underlying mechanisms of MDD, is likely greatly improved by the adoption of the refinements that have been identified over the intervening years since the model was first introduced.

## Supplementary information

ESM 1 (DOCX 262 kb)

ESM 2 (XLSX 107 kb)

ESM 3 (PDF 928 kb)

## References

[CR1] Airaksinen KE (1999). Autonomic mechanisms and sudden death after abrupt coronary occlusion. Ann Med.

[CR2] Akimoto H, Oshima S, Sugiyama T, Negishi A, Nemoto T, Kobayashi D (2019). Changes in brain metabolites related to stress resilience: Metabolomic analysis of the hippocampus in a rat model of depression. Behav Brain Res.

[CR3] Alter M, Rubin D, Ramsey K, Halpern R, Stephan D, Abbott L (2008). Variation in the large-scale organization of gene expression levels in the hippocampus relates to stable epigenetic variability in behavior. PLoS One.

[CR4] Anisman H, Matheson K (2005). Stress, depression and anhedonia: caveats concerning animal models. Neurosci Biobehav Rev.

[CR5] Antoniuk S, Bijata M, Ponimaskin E, Wlodarczyk J (2019). Chronic unpredictable mild stress for modeling depression in rodents: Meta-analysis of model reliability. Neurosci Biobehav Rev.

[CR6] Armario A, Gavaldà A, Martí J (1995). Comparison of the behavioural and endocrine response to forced swimming stress in five inbred strains of rats. Psychoneuroendocrinology..

[CR7] Avery DH, Shah SH, Eder DN, Wildschiødtz G (1999). Nocturnal sweating and temperature in depression. Acta Psychiatr Scand.

[CR8] Baglioni C, Battagliese G, Feige B, Spiegelhalder K, Nissen C, Voderholzer U, Lombardo C, Riemann D (2011). Insomnia as a predictor of depression: a meta-analytic evaluation of longitudinal epidemiological studies. J Affect Disord.

[CR9] Bai M, Zhu XZ, Zhang Y, Zhang S, Zhang L, Xue L, Yi JY, Yao SQ, Zhang XW (2012) Abnormal hippocampal BDNF and miR-16 expression is associated with depression-like behaviors induced by stress during early life. PLoS One 7 10.1371/journal.pone.0046921 PMC3466179 23056528

[CR10] Baker SL, Kentner AC, Konkle AT, Santa-Maria Barbagallo L, Bielajew C (2006). Behavioral and physiological effects of chronic mild stress in female rats. Physiol Behav.

[CR11] Banasr M, Duman RS (2008). Glial loss in the prefrontal cortex is sufficient to induce depressive-like behaviors. Biol Psychiatry.

[CR12] Banasr M, Valentine GW, Li XY, Gourley SL, Taylor JR, Duman RS (2007). Chronic unpredictable stress decreases cell proliferation in the cerebral cortex of the adult rat. Biol Psychiatry.

[CR13] Banasr M, Chowdhury GMI, Terwilliger R, Newton SS, Duman RS, Behar KL, Sanacora G (2010). Glial pathology in an animal model of depression: reversal of stress-induced cellular, metabolic and behavioral deficits by the glutamate modulating drug riluzole. Mol Psychiatry.

[CR14] Barker EL, Kimmel HL, Blakely RD (1994). Chimeric human and rat serotonin transporters reveal domains involved in recognition of transporter ligands. Mol Pharmacol.

[CR15] Barnes SA, Der-Avakian A, Markou A (2014). Anhedonia, avolition, and anticipatory deficits: assessments in animals with relevance to the negative symptoms of schizophrenia. Eur Neuropsychopharmacol.

[CR16] Baune BT, Stuart M, Gilmour A, Wersching H, Heindel W, Arolt V, Berger K (2012). The relationship between subtypes of depression and cardiovascular disease: a systematic review of biological models. Transl Psychiatry.

[CR17] Bekris S, Antoniou K, Daskas S, Papadopoulou-Daifoti Z (2005). Behavioural and neurochemical effects induced by chronic mild stress applied to two different rat strains. Behav Brain Res.

[CR18] Belovicova K, Bogi E, Csatlosova K, Dubovicky M (2017). Animal tests for anxiety-like and depression-like behavior in rats. Interdiscip Toxicol.

[CR19] Bergström A, Jayatissa M, Thykjaer T, Wiborg O (2007). Molecular pathways associated with stress resilience and drug resistance in the chronic mild stress rat model of depression: a gene expression study. J Mol Neurosci.

[CR20] Berry A, Bellisario V, Capoccia S, Tirassa P, Calza A, Alleva E (2012). Social deprivation stress is a triggering factor for the emergence of anxiety- and depression-like behaviours and leads to reduced brain BDNF levels in C57BL/6J mice. Psychoneuroendocrinology.

[CR21] Bertoglio L, Carobrez A (2002). Behavioral profile of rats submitted to session 1-session 2 in the elevated plus-maze during diurnal/nocturnal phases and under different illumination conditions. Behav Brain Res.

[CR22] Bessa JM, Ferreira D, Melo I, Marques F, Cerqueira JJ, Palha JA, Almeida OFX, Sousa N (2009). The mood improving actions of antidepressants do not depend on neurogenesis but are associated with neuronal remodeling. Mol Psychiatry.

[CR23] Bessa J, Morais M, Marque F, Pinto L, Palha J, Almeida O, Sousa N (2013). Stress-induced anhedonia is associated with hypertrophy of medium spiny neurons of the nucleus accumbens. Transl Psychiatry.

[CR24] Billman G, Schwartz P, Stone H (1982). Baroreceptor reflex control of heart rate: a predictor of sudden cardiac death. Circulation..

[CR25] Bisgaard C, Jayatissa M, Enghild J, Sanchéz C, Artemychyn R, Wiborg O (2007). Proteomic investigation of the ventral rat hippocampus links DRP-2 to escitalopram treatment resistance and SNAP to stress resilience in the chronic mild stress model of depression. J Mol Neurosci.

[CR26] Bogdanova O, Kanekar S, D’Anci K, Renshaw P (2013). Factors influencing behavior in the forced swim test. Physiol Behav.

[CR27] Bondi CO, Rodriguez G, Gould GG, Frazer A, Morilak DA (2008). Chronic unpredictable stress induces a cognitive deficit and anxiety-like behavior in rats that is prevented by chronic antidepressant drug treatment. Neuropsychopharmacology.

[CR28] Borsini F (2012). Models for depression in drug screening and preclinical studies: future directions. World J Pharmacol.

[CR29] Bortolato M, Mangieri RA, Fu J, Kim JH, Arguello O, Duranti A, Tontini A, Mor M, Tarzia G, Piomelli D (2007). Antidepressant-like activity of the fatty acid amide hydrolase inhibitor URB597 in a rat model of chronic mild stress. Biol Psychiatry.

[CR30] Boulle F, Massart R, Stragier E, Païzanis E, Zaidan L, Marday S, Gabriel C, Mocaer E, Mongeau R, Lanfumey L (2014). Hippocampal and behavioural dysfunctions in a mouse model of environmental stress: normalization by agomelatine. Transl Psychiatry.

[CR31] Bouzinova EV, Møller-Nielsen N, Boedtkjer DB, Broegger T, Wiborg O, Aalkjaer C, Matchkov VV (2012). Chronic mild stress-induced depression-like symptoms in rats and abnormalities in catecholamine uptake in small arteries. Psychosom Med.

[CR32] Branchey L, Weinberg U, Branchey M, Linkowski P, Mendlewicz J (1982). Simultaneous study of 24-hour patterns of melatonin and cortosil secretion in depressed patients. Neuropsychobiology..

[CR33] Brennan K, Roberts D, Anisman H, Merali Z (2001). Individual differences in sucrose consumption in the rat: motivational and neurochemical correlates of hedonia. Psychopharmacology..

[CR34] Cabib S (1997). What is mild in mild stress?. Psychopharmacology..

[CR35] Chad EB, Jason MD, Michael JP, Brian JP, Ru S, Zia R, Karen C, Melissa TM, Tarek AS, Jeffrey DK, Brendan B, Garth TW (2010) Depression-like phenotype following chronic CB1 receptor antagonism. Neurobiol Dis 2:148-155 10.1016/j.nbd.2010.03.020 20381618

[CR36] Castagne V, Porsolt R, Moser P (2009). Use of latency to immobility improves detection of antidepressant-like activity in the behavioral despair test in the mouse. Eur J Pharmacol.

[CR37] Chang CH, Grace AA (2014). Amygdala-ventral pallidum pathway decreases dopamine activity after chronic mild stress in rats. Biol Psychiatry.

[CR38] Chaouloff F, Kulikov A, Sarrieau A, Castanon N, Mormede P (1995). Male Fischer 344 and Lewis rats display differences in locomotor reactivity, but not in anxiety-related behaviours: relationship with the hippocampal serotonergic system. Brain Res.

[CR39] Chaturvedi SK (2020). Covid-19, coronavirus and mental health rehabilitation at times of crisis. J Psychosoc Rehabil Ment Health.

[CR40] Cheeta S, Ruigt G, van Proosdij J, Willner P (1997). Changes in sleep architecture following chronic mild stress. Biol Psychiatry.

[CR41] Chen AT, Malmstrom T, Nasrallah HA (2018). Body temperature rises following improvement of depression with ECT. Ann Clin Psychiatry.

[CR42] Christiansen S, Bouzinova EV, Palme R, Wiborg O (2012). Circadian activity of the hypothalamic-pituitary-adrenal axis is differentially affected in the rat chronic mild stress model of depression. Stress-the International Journal on the Biology of Stress.

[CR43] Christiansen S, Bouzinova E, Fahrenkrug J, Wilborg O (2016) Altered Expression Pattern of Clock Genes in a Rat Model of Depression. Int J Neuropsychopharmacol 19:pyw061 10.1093/ijnp/pyw061 PMC5137278 27365111

[CR44] Cline B, Costa-Nunes J, Cespuglio R, Markova N, Santos A, Bukhman Y, Kubatiev A, Steinbusch H, Lesch K, Strekalova T (2015). Dicholine succinate, the neuronal insulin sensitizer, normalizes behavior, REM sleep, hippocampal pGSK3 beta and mRNAs of NMDA receptor subunits in mouse models of depression. Front Behav Neurosci.

[CR45] Cole B, Koob G (1994). Corticotropin-releasing factor and schedule-induced polydipsia. Pharmacol Biochem Behav.

[CR46] Commons KG, Cholanians AB, Babb JA, Ehlinger DG (2017). The rodent forced swim test measures stress-coping strategy, not depression-like behavior. ACS Chem Neurosci.

[CR47] Costa-Nunes JP, Gorlova A, Pavlov D, Cespuglio R, Gorovaya A, Proshin A, Umriukhin A, Ponomarev ED, Kalueff AV, Strekalova T, Schroeter CA (2020). Ultrasound stress compromises the correlates of emotional-like states and brain AMPAR expression in mice: effects of antioxidant and anti-inflammatory herbal treatment. Stress..

[CR48] Couch Y, Anthony D, Dolgov O, Revischin A, Festoff B, Santos A, Steinbusch H, Strekalova T (2013). Microglial activation, increased TNF and SERT expression in the prefrontal cortex define stress behaviour in mice susceptible to anhedonia. Brain Behav Immun.

[CR49] Couch Y, Trofimov A, Markova N, Nikolenko V, Steinbusch H, Chekhonin V, Schroeter C, Lesch K, Anthony D, Strekalova T (2016). Low-dose lipopolysaccharide (LPS) inhibits aggressive and augments depressive behaviours in a chronic mild stress model in mice. J Neuroinflammation.

[CR50] Coudereau J, Stain F, Drion N, Sandouk P, Monier C, Debray M, Scherrmann JM, Bourre J, Frances H (1999). Effect of social isolation on the metabolism of morphine and its passage through the blood-brain barrier and on consumption of sucrose solutions. Psychopharmacology.

[CR51] Crestani C, Tavares R, Guimarães F, Correa F, Joca S, Resstel L (2011). Chronic fluoxetine treatment alters cardiovascular functions in unanesthetized rats. Eur J Pharmacol.

[CR52] Cryan J, Slattery D (2007). Animal models of mood disorders: recent developments. Curr Opin Psychiatry.

[CR53] Cryan J, Markou A, Lucki I (2002). Assessing antidepressant activity in rodents: recent developments and future needs. Trends Pharmacol Sci.

[CR54] Cryan JF, Valentino RJ, Lucki I (2005). Assessing substrates underlying the behavioral effects of antidepressants using the modified rat forced swimming test. Neurosci Biobehav Rev.

[CR55] Cudnoch-Jedrzejewska A, Szczepanska-Sadowska E, Dobruch J, Gomolka R, Puchalska L (2010). Brain vasopressin V-1 receptors contribute to enhanced cardiovascular responses to acute stress in chronically stressed rats and rats with myocardial infarcton. Am J Phys Regul Integr Comp Phys.

[CR56] Czéh B, Vardya I, Varga Z, Febbraro F, Csabai D, Martis LS et al (2018) Long-term stress disrupts the structural and functional integrity of GABAergic neuronal networks in the medial prefrontal cortex of rats. Front Cell Neurosci 12 10.3389/fncel.2018.00148 PMC6020798 29973870

[CR57] Dalla C, Antoniou K, Drossopoulou G, Xagoraris M, Kokras N, Sfikakis A, Papadopoulou-Daifoti Z (2005). Chronic mild stress impact: are females more vulnerable?. Neuroscience.

[CR58] Dalla C, Pitychoutis PM, Kokras N, Papadopoulou-Daifoti Z (2011). Sex differences in response to stress and expression of depressive-like behaviours in the rat. Curr Top Behav Neurosci.

[CR59] Daquila P, Brain P, Willner P (1994). Effects of chronic mild stress on performance in behavioral tests relevant to anxiety and depression. Physiol Behav.

[CR60] de Kloet E, Molendijk M (2016). Coping with the Forced Swim Stressor: Towards Understanding an Adaptive Mechanism. Neural Plast.

[CR61] Delgado R, Campo A, Henningsen K, Verhoye M, Poot D, Dijkstra J, Van Audekerke J, Benveniste H, Sijbers J, Wiborg O, Van der Linden A (2011). Magnetic resonance imaging and spectroscopy reveal differential hippocampal changes in anhedonic and resilient subtypes of the chronic mild stress rat model. Biol Psychiatry.

[CR62] Dell’Osso L, Massimetti G, Conversano C, Bertelloni C, Carta M, Ricca V, Carmassi (2014). Alterations in circadian/seasonal rhythms and vegetative functions are related to suicidality in DSM-5 PTSD. BMS Psychiatry.

[CR63] Demaestri C, Brenhouse HC, Honeycutt JA (2019). 22 kHz and 55 kHz ultrasonic vocalizations differentially influence neural and behavioral outcomes: Implications for modeling anxiety via auditory stimuli in the rat. Behav Brain Res.

[CR64] Demin KA, Sysoev M, Chernysh MV, Savva AK, Koshiba M, Wappler-Guzzetta EA, Song C, De Abreu MS, Leonard B, Parker MO, Harvey BH, Tian L, Vasar E, Strekalova T, Amstislavskaya TG, Volgin AD, Alpyshov ET, Wang D, Kalueff AV (2019). Animal models of major depressive disorder and the implications for drug discovery and development. Expert Opin Drug Discovery.

[CR65] Der-Avakian A, Michelle S, Mazei-Robison MS, Kesby JP, Nestler EJ, Markou A (2014). Enduring deficits in brain reward function after chronic social defeat in rats: susceptibility, resilience, and antidepressant response. Biol Psychiatry.

[CR66] Diamantopoulou A, Kalpachidou T, Aspiotis G, Gampierakis I, Stylianopoulou F, Stamatakis A (2018). An early experience of mild adversity involving temporary denial of maternal contact affects the serotonergic system of adult male rats and leads to a depressive-like phenotype and inability to adapt to a chronic social stress. Physiol Behav.

[CR67] Domeney A, Feldon J (1998). The disruption of prepulse inhibition by social isolation in the Wistar rat: how robust is the effect?. Pharmacol Biochem Behav.

[CR68] Dubovsky SL (2018). What Is New about New Antidepressants. Psychother Psychosom.

[CR69] Duclot F, Hollis F, Darcy MJ, Kabbaj M (2011). Individual differences in novelty-seeking behavior in rats as a model for psychosocial stress-related mood disorders. Physiol Behav.

[CR70] Ellenbroek B, Youn J (2016). Rodent models in neuroscience research: is it a rat race? Disease Models &amp. Mechanisms..

[CR71] Fan CQ, Song QQ, Wang P, Li Y, Yang M, Yu SY (2018) Neuroprotective effects of Ginsenoside-Rg1 against depression-like behaviors via suppressing glial activation, synaptic deficits, and neuronal apoptosis in rats. Front Immunol 9 10.3389/fimmu.2018.02889 PMC6292928 30581440

[CR72] Faron-Górecka A, Kuśmider M, Kolasa M, Żurawek D, Szafran-Pilch K, Gruca P, Pabian P, Solich J, Papp M, Dziedzicka-Wasylewska M (2016). Chronic mild stress alters the somatostatin receptors in the rat brain. Psychopharmacology.

[CR73] Febbraro F, Svenningsen K, Tran TP, Wiborg O (2017) Neuronal substrates underlying stress resilience and susceptibility in rats. PLoS One 12 10.1371/journal.pone.0179434 PMC5473563 28622391

[CR74] Feder A, Nestler EJ, Charney DS (2009). Psychobiology and molecular genetics of resilience. Nat Rev Neurosci.

[CR75] Ferreira MF, Castanheira L, Sebastião AM, Telles-Correia D (2018). Depression assessment in clinical trials and pre-clinical tests: a critical review. Curr Top Med Chem.

[CR76] Finnell JE, Muniz BL, Padi AR, Lombard CM, Moffitt CM, Wood CS (2018). Essential role of ovarian hormones in susceptibility to the consequences of witnessing social defeat in female rats. Biol Psychiatry.

[CR77] Forbes NF, Stewart CA, Matthews K, Reid IC (1996). Chronic mild stress and sucrose consumption: validity as a model of depression. Physiol Behav.

[CR78] Franceschelli A, Herchick S, Thelen C, Papadopoulou-Daifoti Z, Pitychoutis P (2014). Sex differences in the chronic mild stress model of depression. Behav Pharmacol.

[CR79] Franklin TC, Wohleb ES, Zhang Y, Fogaca M, Hare B, Duman RS (2018). Persistent increase in microglial RAGE contributes to chronic stress-induced priming of depressive-like behavior. Biol Psychiatry.

[CR80] Frey A, Popp S, Post A, Langer S, Lehmann M, Hofmann U, Sirén AL, Hommers L, Schmitt A, Strekalova T, Ertl G, Lesch KP (2014). Experimental heart failure causes depression-like behavior together with differential regulation of inflammatory and structural genes in the brain. Front Behav Neurosci.

[CR81] Gambarana C, Scheggi S, Tagliamonte A, Tolu P, De Montis M (2001). Animal models for the study of antidepressant activity. Brain Res Protocol.

[CR82] Garza JC, Guo M, Zhang W, Lu XY (2012). Leptin restores adult hippocampal neurogenesis in a chronic unpredictable stress model of depression and reverses glucocorticoid-induced inhibition of GSK-3 beta/beta-catenin signaling. Mol Psychiatry.

[CR83] Gauthier G, Mucha L, Shi S, Guerin A (2019). Economic burden of relapse/recurrence in patients with major depressive disorder. J Drug Assess.

[CR84] Gizowski C, Zaelzer C, Bourque CW (2016). Clock-driven vasopressin neurotransmission mediates anticipatory thirst prior to sleep. Nature..

[CR85] Glendinning J, Gresack J (2002). A high-throughput screening procedure for identifying mice with aberrant taste and oromotor function. Chem Senses.

[CR86] Goh KK, Chang SC, Chen CH, Lu ML (2020). Therapeutic Strategies for Treatment-resistant Depression: State of the Art and Future Perspectives. Curr Pharm Des.

[CR87] Gorinski N, Bijata M, Prasad S, Wirth A, Abdel Galil D, Zeug A, Bazovkina D, Kondaurova E, Kulikova E, Ilchibaeva T, Zareba-Koziol M, Papaleo F, Scheggia D, Kochlamazashvili G, Dityatev A, Smyth I, Krzystyniak A, Wlodarczyk J, Richter DW (2019). Attenuated palmitoylation of serotonin receptor 5-HT1A affects receptor function and contributes to depression-like behaviors. Nat Commun.

[CR88] Gorka Z, Moryl E, Papp M (1996). Effect of chronic mild stress on circadian rhythms in the locomotor activity in rats. Pharmacol Biochem Behav.

[CR89] Grant MJ, Booth A (2009). A typology of reviews: an analysis of 14 review types and associated methodologies. Health Inf Libr J.

[CR90] Greene J, Banasr M, Lee B, Warner-Schmidt J, Duman R (2009) Vascular endothelial growth factor signaling is required for the behavioral actions of antidepressant treatment: pharmacological and cellular characterization. Neuropsychopharmacology 34(11):2459–2468. 10.1038/npp.2009.68 10.1038/npp.2009.68 PMC3694572 19553916

[CR91] Grippo A (2009). Mechanisms underlying altered mood and cardiovascular dysfunction: the value of neurobiological and behavioral research with animal models. Neurosci Biobehav Rev.

[CR92] Grippo AJ, Johnson AK (2002) Biological mechanisms in the relationship between depression and heart disease. Neurosci Biobehav Rev 8:941-962 10.1016/s0149-7634(03)00003-4 12667498

[CR93] Grippo AJ, Beltz TG, Johnson AK (2003) Behavioral and cardiovascular changes in the chronic mild stress model of depression. Physiol Behav 4-5:703-710 10.1016/s0031-9384(03)00050-7 12782226

[CR94] Grippo AJ, Moffitt JA, Sgoifo A, Jepson AJ, Bates SL, Chandler DL, McNeal N, Preihs K (2012) The integration of depressive behaviors and cardiac dysfunction during an operational measure of depression: investigating the role of negative social experiences in an animal model. Psychosom Med 6:612-619 10.1097/PSY.0b013e31825ca8e5 PMC3392416 22753634

[CR95] Gronli J, Murison R, Bjorvath B, Sorensen E, Portas C, Ursin R (2004). Chronic mild stress affects sucrose intake and sleep in rats. Behav Brain Res.

[CR96] Gronli J, Dagestad G, Milde A, Murison R, Bramham C (2012). Post-transcriptional effects and interactions between chronic mild stress and acute sleep deprivation: regulation of translation factor and cytoplasmic polyadenylation element-binding protein phosphorylation. Behav Brain Res.

[CR97] Guesdon B, Messaoudi M, Lefranc-Millot C, Fromentin G, Tome D, Even P (2006). A tryptic hydrolysate from bovine milk alphaS1-casein improves sleep in rats subjected to chronic mild stress. Peptides..

[CR98] Hagan JJ, Hatcher JP (1997). Revised CMS model. Psychopharmacology.

[CR99] Hamilton M (1967). Development of a rating scale for primary depressive illness. Br J Clin Psychol.

[CR100] Hao Y, Hu Y, Wang H, Paudel D, Xu Y, Zhang B (2019) The Effect Of Fluvoxamine On Sleep Architecture Of Depressed Patients With Insomnia: An 8-Week, Open-Label, Baseline-Controlled Study. Nat Sci Sleep 11:291-300 10.2147/NSS.S220947 PMC6839582 31807102

[CR101] Harkin A, Connor T, O’Donnell KJ (2002). Physiological and behavioral responses to stress: what does a rat find stressful?. Lab Anim.

[CR102] Harriman (1976). Preferences by northern grasshopper mice for solutions of sugars, acids, and salts in Richter-type drinking tests. J Gen Psychol.

[CR103] Harris R, Zhou J, Youngblood B, Smagin G, Ryan D (1997). Failure to change exploration or saccharin preference in rats exposed to chronic mild stress. Physiol Behav.

[CR104] Harro J (2013). Animal models of depression vulnerability. Current Topics in Behavioral.

[CR105] Harro J (2019). Animal models of depression: pros and cons. Cell Tissue Res.

[CR106] Harro J, Kiive E (2011). Droplets of black bile? Development of vulnerability and resilience to depression in young age. Psychoneuroendocrinology..

[CR107] Hasin DS, Sarvet AL, Meyers JL, Saha TD, Ruan WJ, Stohl M, Grant BF (2018). Epidemiology of adult DSM-5 major depressive disorder and its specifiers in the United States. JAMA Psychiatry.

[CR108] Hata T, Nishikawa H, Itoh E, Watanabe A (1999). Depressive state with anxiety in repeated cold-stress mice in forced swimming tests. Jpn J Pharmacol.

[CR109] Hatcher J, Bell N, Reed J, Hagan J (1997). Chronic mild stress-induced reductions in saccharin intake depend upon feeding status. J Psychopharmacol.

[CR110] Heine VM, Maslam S, Zareno J, Joels M, Lucassen PJ (2004). Suppressed proliferation and apoptotic changes in the rat dentate gyrus after acute and chronic stress are reversible. Eur J Neurosci.

[CR111] Henningsen K, Andreasen JT, Bouzinova EV, Jayatissa MN, Jensen MS, Redrobe JP, Wiborg O (2009). Cognitive deficits in the rat chronic mild stress model for depression: relation to anhedonic-like responses. Behav Brain Res.

[CR112] Henningsen K, Palmfeldt J, Christiansen S, Baiges I, Bak S, Jensen ON et al (2012) Candidate Hippocampal Biomarkers of Susceptibility and Resilience to Stress in a Rat Model of Depression. Mol Cell Proteomics 11 10.1074/mcp.M111.016428 PMC3394954 22311638

[CR113] Henningsen K, Woldby D, Wiborg O (2013). Electroconvulsive stimulation reverses anhedonia and cognitive impairments in rats exposed to chronic mild stress. Eur Neuropsychopharmacol.

[CR114] Herrera-Pérez JJ, Martínez-Mota L, Chavira R, Fernández-Guasti A (2012). Testosterone prevents but not reverses anhedonia in middle-aged males and lacks an effect on stress vulnerability in young adults. Horm Behav.

[CR115] Heyman SE (2007). How mice cope with stressful social situations. Cell..

[CR116] Hill MN, Patel S, Carrier EJ, Rademacher DJ, Ormerod BK, Hillard CJ (2005). Downregulation of endocannabinoid signaling in the hippocampus following chronic unpredictable stress. Neuropsychopharmacology.

[CR117] Høifødt R, Waterloo K, Wang CEA, Eisemann M, Figenschau Y, Halvorsen M (2019). Cortisol levels and cognitive profile in major depression: A comparison of currently and previously depressed patients. Psychoneuroendocrinology..

[CR118] Holmes PV (2003). Rodent models of depression: reexamining validity without anthropomorphic inference. Crit Rev Neurobiol.

[CR119] Houwing DJ, Ramsteijn AS, Riemersma IW, Olivier JDA (2019). Maternal separation induces anhedonia in female heterozygous serotonin transporter knockout rats. Behav Brain Res.

[CR120] Hu J, Zhou Q, Yang S, Chen H, Zhang L, Yan Y, Hou Y (2011). Metal stress-induced arrhythmia and thoracic spinal cord 1-5 nerve remodeling and myocardial electrophysiological remodeling in rats. Zhonghua Xin Xue Guan Bing Za Zhi.

[CR121] Hu C, Luo Y, Wang H, Kuang S, Liang G, Yang Y, Mai S, Yang J (2017). Re-evaluation of the interrelationships among the behavioral tests in rats exposed to chronic unpredictable mild stress. PLoS One.

[CR122] Igarashi E, Takeshita S (1995). Effects of illumination and handling upon rat open field activity. Physiol Behav.

[CR123] Insel T (2009). Disruptive insights in psychiatry: transforming a clinical discipline. J Clin Invest.

[CR124] Insel T, Sahakian B (2012). Drug research: a plan for mental illness. Nature.

[CR125] Jakovcevski M, Schachner M, Morellini F (2008). Individual variability in the stress response of C57BL/6J male mice correlates with trait anxiety. Genes Brain Behav.

[CR126] Jayatissa MN, Bisgaard C, Tingstrom A (2006). Hippocampal cytogenesis correlates to escitalopram-mediated recovery in a chronic mild stress rat model of depression. Neuropsychopharm..

[CR127] Jayatissa M, Bisgaard C, West M, Wiborg O (2008). The number of granule cells in rat hippocampus is reduced after chronic mild stress and re-established after chronic escitalopram treatment. Neuropharmacology..

[CR128] Jayatissa M, Henningsen K, West MJ, Wiborg O (2009). Decreased cell proliferation in the dentate gyrus does not associate with development of anhedonic-like symptoms in rats. Brain Res.

[CR129] Jayatissa MN, Henningsen K, Nikolajsen G, West MJ, Wiborg O (2010). A reduced number of hippocampal granule cells does not associate with an anhedonia-like phenotype in a rat chronic mild stress model of depression. Stress..

[CR130] Jensen TL, Kiersgaard MK, Sørensen DB, Mikkelsen LF (2013). Fasting of mice: a review. Lab Anim.

[CR131] Jia Y, Liu L, Sheng C, Cheng Z, Cui L, Li M, Zhao Y, Shi T, Li F, Chen L, Yau TO (2019). Increased serum levels of cortisol and inflammatory cytokines in people with depression. J Nerv Ment Dis.

[CR132] Kafetzopoulos V, Kokras N, Sotiropoulos I, Oliveira JF, Leite-Almeida H, Vasalou A, Sardinha VM, Papadopoulou-Daifoti Z, Almeida OFX, Antoniou K, Sousa NC (2018). The nucleus reuniens: a key node in the neurocircuitry of stress and depression. Mol Psychiatry.

[CR133] Kant G, Baumann R (1993). Effects of chronic stress and time of day on preference for sucrose. Physiol Behav.

[CR134] Karson A, Demirtas T, Bayramgurler D, Balci F, Utkan T (2013). Chronic Administration of Infliximab (TNF-alpha inhibitor) decreases depression and anxiety-like behaviour in rat model of chronic mild stress. Basic Clin Pharmacol Toxicol.

[CR135] Katz R (1981). Animal model and human depressive disorders. Neorosci Behav Rev.

[CR136] Katz R (1982). Animal model of depression: Pharmacological sensitivity of hedonic deficit. Pharmacol Biochem Behav.

[CR137] Katz R (1984). Effects of zometapine, a structurally novel antidepressant, in animal modeldepression. Pharmacol Biochem Behav.

[CR138] Kelliher P, Connor TJ, Harkin A, Sanchez C, Kelly JP, Leonard BE (2000). Varying responses to the rat forced-swim test under diurnal and nocturnal conditions. Physiol Behav.

[CR139] Kessler RC, Bromet EJ (2013). The epidemiology of depression across cultures. Annu Rev Public Health.

[CR140] Kessler RC, Chiu WT, Demler O (2005). Prevalence, severity and comorbidity of 12-month DSM-IV disorders in the National Comorbidity Survey Replication. Arch Gen Psychiatry.

[CR141] Klein DF (1974). Endogenomorphic depression. A conceptual and terminological revision. Arch Gen Psychiatry.

[CR142] Klenerova V, Jurcovicova J, Kaminsky O (2003). Combined restraint and cold stress in rats: effects on memory processing in passive avoidance task and on plasma levels of ACTH and corticosterone. Behav Brain Res.

[CR143] Kõiv K, Vares M, Kroon C, Metelitsa M, Tiitsaar K, Laugus K, Jaako K, Harro J (2019). Effect of chronic variable stress on sensitization to amphetamine in high and low sucrose-consuming rats. J Psychopharmacol.

[CR144] Kolasa M, Faron-Gorecka A, Kusmider M, Szafran-Plich K, Solich J, Zurawek D, Gruca P, Papp M, Dziedzicka-Wasylewska M (2014). Differential stress response in rats subjected to chronic mild stress is accompanied by changes in CRH-family gene expression at the pituitary level. Peptides..

[CR145] Kompagne H, Bárdos G, Szénási G, Gacsályi I, Hársing LG, Lévay G (2008). Chronic mild stress generates clear depressive but ambiguous anxiety-like behaviour in rats. Behav Brain Res.

[CR146] Koo JW, Duman RS (2008). IL-1 beta is an essential mediator of the antineurogenic and anhedonic effects of stress. Proc Natl Acad Sci U S A.

[CR147] Koprdova R, Bogi E, Belovicova K, Sedlackova N, Okuliarova M, Ujhazy E, Mach M (2016). Chronic unpredictable mild stress paradigm in male Wistar rats: effect on anxiety- and depressive-like behavior. Neuro Endocrinol Lett.

[CR148] Kreisel T, Frank MG, Licht T, Reshef R, Ben-Menachem-Zidon O, Baratta MV, Maier SF, Yirmiya R (2014). Dynamic microglial alterations underlie stress-induced depressive-like behavior and suppressed neurogenesis. Mol Psychiatry.

[CR149] Krimm R, Nejad M, Smith J, Miller I, Beidler L (1987). The effect of bilateral sectioning of the chorda tympani and the greater superficial petrosal nerves on the sweet taste in the rat. Physiol Behav.

[CR150] Krishnan V, Han MH, Graham DL, Berton O, Renthal W, Russo SJ (2007). Molecular adaptations underlying susceptibility and resistance to social defeat in brain reward regions. Cell..

[CR151] Labaka A, Gómez-Lazaro E, Goñi-Balentziaga O, Pérez-Tejada J, Vegas O, Garmendia L (2021). Venlafaxine reduces the striatal il6/il10 ratio and increases hippocampal GR expression in female mice subjected to chronic social instability stress. Stress..

[CR152] Landgraf R (2003). Animal models of anxiety. Stress..

[CR153] Larsen MH, Mikkelsen JD, Hay-Schmidt A, Sandi C (2010). Regulation of brain-derived neurotrophic factor (BDNF) in the chronic unpredictable stress rat model and the effects of chronic antidepressant treatment. J Psychiatr Res.

[CR154] Lesch K, Mössner R (2006). Inactivation of 5HT transport in mice: modeling altered 5HT homeostasis implicated in emotional dysfunction, affective disorders, and somatic syndromes. Handb Exp Pharmacol.

[CR155] Levine R (1967). Genetic relationships, choice models, and sucrose preference behaviour in mice. Nature..

[CR156] Li NX, Liu RJ, Dwyer JM, Banasr M, Lee B, Son H (2011). Glutamate N-methyl-D-aspartate receptor antagonists rapidly reverse behavioral and synaptic deficits caused by chronic stress exposure. Biol Psychiatry.

[CR157] Li Y, Wang HL, Wang XP, Liu ZC, Wan QR, Wang GH (2014). Differential expression of hippocampal EphA4 and ephrinA3 in anhedonic-like behavior, stress resilience, and antidepressant drug treatment after chronic unpredicted mild stress. Neurosci Lett.

[CR158] Li SX, Han Y, Xu LZ, Yuan K, Zhang RX, Sun CY, Xu DF, Yuan M, Deng JH, Meng SQ, Gao XJ, Wen Q, Liu LJ, Zhu WL, Xue YX, Zhao M, Shi J, Lu L (2018). Uncoupling DAPK1 from NMDA receptor GluN2B subunit exerts rapid antidepressant-like effects. Mol Psychiatry.

[CR159] Liu B, Xu C, Wu X, Liu F, Du Y, Sun J, Tao J, Dong J (2015). Icariin exerts an antidepressant effect in an unpredictable chronic mild stress model of depression in rats and is associated with the regulation of hippocampal neuroinflammation. Neuroscience.

[CR160] Liu Y, Zhao J, Guo W (2018). Emotional roles of mono-aminergic neurotransmitters in major depressive disorder and anxiety disorders. Front Psychol.

[CR161] Lu XY, Kim CS, Frazer A, Zhang W (2006). Leptin: A potential novel antidepressant. Proc Natl Acad Sci U S A.

[CR162] Lu YX, Ho CS, McIntyre RS, Wang W, Ho RC (2018). Effects of vortioxetine and fluoxetine on the level of Brain Derived Neurotrophic Factors (BDNF) in the hippocampus of chronic unpredictable mild stress-induced depressive rats. Brain Res Bull.

[CR163] Lucas G, Rymar VV, Du J, Mnie-Filali O, Bisgaard C, Manta S, Lambas-Senas L, Wiborg O, Haddjeri N, Pineyro G, Sadikot AF, Debonnel G (2007). Serotonin(4) (5-HT4) receptor agonists are putative antidepressants with a rapid onset of action. Neuron.

[CR164] Luo DD, An SC, Zhang X (2008). Involvement of hippocampal serotonin and neuropeptide Y in depression induced by chronic unpredicted mild stress. Brain Res Bull.

[CR165] Markova N, Bazhenova N, Anthony D, Vignisse J, Svistunov A, Lesch K, Bettendorff ST (2017). Thiamine and benfotiamine improve cognition and ameliorate GSK-3β-associated stress-induced behaviours in mice. Prog Neuro-Psychopharmacol Biol Psychiatry.

[CR166] Martis LS, Brision C, Holmes MC, Wiborg O (2018). Resilient and depressive-like rats show distinct cognitive impairments in the touchscreen paired-associates learning (PAL) task. Neurobiol Learn Mem.

[CR167] Matchkov VV, Kravtsova VV, Wiborg O, Aalkjaer C, Bouzinova EV (2015). Chronic selective serotonin reuptake inhibition modulates endothelial dysfunction and oxidative state in rat chronic mild stress model of depression. Am J Phys Regul Integr Comp Phys.

[CR168] Matthews K, Forbes N, Reid I (1995). Sucrose consumption as an hedonic measure following chronic unpredictable mild stress. Physiol Behav.

[CR169] McArthur R, Borsini F (2006). Animal models of depression in drug discovery: a historical perspective. Pharmacol Biochem Behav.

[CR170] McLaughlin R, Hill M, Dang S, Wainwright S, Galea L, Hillard C, Gorzalka B (2013). Upregulation of CB(1) receptor binding in the ventromedial prefrontal cortex promotes proactive stress-coping strategies following chronic stress exposure. Behav Brain Res.

[CR171] Meerlo P, Sgoifo A, De Boer SF, Koolhaas J (1999). Long-lasting consequences of a social conflict in rats: behavior during the interaction predicts subsequent changes in daily rhythms of heart rate, temperature, and activity. Behav Neurosci.

[CR172] Mill J, Petronis A (2007). Molecular studies of major depressive disorder: the epigenetic perspective. Mol Psychiatry.

[CR173] Moeler H-J (2017). Why are new antidepressants failing to make the grade for approval?. Exp Opinion Pharm.

[CR174] Molendijk ML, de Kloet ER (2019). Coping with the forced swim stressor: Current state-of-the-art. Behav Brain Res.

[CR175] Moreau J-L (2002). Simulating the anhedonia symptom of depression in animals. Dialogues Clin Neurosci.

[CR176] Moreau JL, Scherschlicht R, Jenck F, Martin JR (1995). Chronic mild stress-induced anhedonia model of depression - sleep abnormalities and curative effects of electroshock treatment. Behav Pharmacol.

[CR177] Morozova A, Zubkov E, Strekalova T, Kekelidze Z, Storozeva Z, Schroeter CA, Bazhenova N, Lesch KP, Cline BH, Chekhonin V (2016). Ultrasound of alternating frequencies and variable emotional impact evokes depressive syndrome in mice and rats. Prog Neuro-Psychopharmacol Biol Psychiatry.

[CR178] Munos B (2009). Lessons from 60 years of pharmaceutical innovation. Nat Rev Drug Discov.

[CR179] Muscat P, Willner P (1992). Suppression of sucrose drinking by chronic mild unpredictable stress: a methodological analysis Neurosci. Biobehav Rev.

[CR180] Nakatake Y, Furuie H, Ukezono M, Yamada M, Yoshizawa K, Yamada M (2020). Indirect exposure to socially defeated conspecifics using recorded video activates the HPA axis and reduces reward sensitivity in mice. Sci Rep.

[CR181] Nechita F, Pirlog MC, Chirita AL (2015). Circadian malfunctions in depression - neurobiological and psychosocial approaches. Romanian J Morphol Embryol.

[CR182] Nestler EJ, Gould E, Manji H, Buncan M, Duman RS, Greshenfeld HK, Hen R, Kester S, Ledehendleer I, Meaney M, Robbins T, Winsky L, Zalcman S (2002). Preclinical models: Status of basic research in depression. Biol Psychiatry.

[CR183] Neumann I, Wegener G, Homberg J, Cohen H, Slattery DA, Zohar J (2011). Animal models of depression and anxiety: What do they tell us about human condition?. Prog Neuro-Psychopharmacol Biol Psychiatry.

[CR184] Nielsen C, Arnt J, Sanchez C (2000). Intracranial self-stimulation and sucrose intake differ as hedonic measures following chronic stress: interstrain and interindividual differences. Behav Brain Res.

[CR185] Nieto-Gonzalez JL, Holm MM, Vardya I, Christensen T, Wiborg O, Jensen K (2015) Presynaptic Plasticity as a Hallmark of Rat Stress Susceptibility and Antidepressant Response. PLoS One 10 10.1371/journal.pone.0119993 PMC4350919 25742132

[CR186] Ohl F, Toschi N, Wigger A, Henniger MS, Landgraf R (2001). Dimensions of emotionality in a rat model of innate anxiety. Behav Neurosci.

[CR187] Pacchiarotti I, Kotzalidis GD, Murru A, Mazzarini L, Rapinesi C, Valentí M, Anmella G, Gomes-da-Costa S, Gimenez A, Llach C, Perugi G, Vieta E, Verdolini N (2020). Mixed features in depression: the unmet needs of diagnostic and statistical manual of mental disorders fifth edition. Psychiatr Clin North Am.

[CR188] Palacios RDY, Verhoye M, Henningsen K, Wiborg O, Van der Linden A (2014) Diffusion kurtosis imaging and high-resolution MRI demonstrate structural aberrations of caudate putamen and amygdala after chronic mild stress. PLoS One 9 10.1371/journal.pone.0095077 PMC3989315 24740310

[CR189] Palmfeldt J, Henningsen K, Eriksen SA, Müller HK, Wiborg O (2016). Protein biomarkers of susceptibility and resilience to stress in a rat model of depression. Mol Cell Neurosci.

[CR190] Papp M (2012). Models of affective illness: chronic mild stress in the rat. Curr Protoc Pharmacol.

[CR191] Papp M, Moryl E (1994). Antidepressant activity of noncompetitive and competitive nmda receptor antagonists in a chronic mild stress model of depression. Eur J Pharmacol.

[CR192] Papp M, Willner P, Muscat R (1991). An animal-model of anhedonia - attenuation of sucrose consumption and place preference conditioning by chronic unpredictable mild stress. Psychopharmacology.

[CR193] Papp M, Nalepa I, Vetulani J (1994). Reversal by imipramine on serotonergic and beta-adrenergic receptor binding in a chronic mild stress model of depression. Eur J Pharmacol.

[CR194] Papp M, Moryl E, Willner P (1996). Pharmacological validation of the chronic mild stress model of depression. Eur J Pharmacol.

[CR195] Papp M, Gruca P, Boyer PA, Mocaër E (2003). Effect of agomelatine in the chronic mild stress model of depression in the rat. Neuropsychopharmacology..

[CR196] Papp M, Gruca P, Lason-Tyburkiewicz M, Willner P (2016). Antidepressant, anxiolytic and procognitive effects of rivastigmine and donepezil in the chronic mild stress model in rats. Psychopharmacology..

[CR197] Park SE, Park D, Song K-I, Seong J-K, Chung S, Youn I (2017). Differential heart rate variability and physiological responses associated with accumulated short- and long-term stress in rodents. Physiol Behav.

[CR198] Pavlov D, Bettendorff L, Gorlova A, Olkhovik A, Kalueff A, Ponomarev E, Inozemtsev A, Chekhonin V, Lesсh K, Anthony D, Strekalova T (2019). Neuroinflammation and aberrant hippocampal plasticity in a mouse model of emotional stress evoked by exposure to ultrasound of alternating frequencies. Prog Neuro-Psychopharmacol Biol Psychiatry.

[CR199] Pawluski J, Valença A, Santos A, Costa-Nunes J, Steinbusch H, Strekalova T (2012). Pregnancy or stress decrease complexity of CA3 pyramidal neurons in the hippocampus of adult female rats. Neuroscience..

[CR200] Pechlivanova D, Tchekalarova J, Nikolov R, Yakimova K (2010). Dose-dependent effects of caffeine on behavior and thermoregulation in a chronic unpredictable stress model of depression in rats. Behav Brain Res.

[CR201] Péquignot R, Dufouil C, Prugger C, Pérès K, Artero S, Tzourio C, Empana J (2016). High Level of Depressive Symptoms at Repeated Study Visits and Risk of Coronary Heart Disease and Stroke over 10 Years in Older Adults: The Three-City Study. J Am Geriatr Soc.

[CR202] Phillips AG, Barr AM (1997). Effects of chronic mild stress on motivation for sucrose: mixed messages. Psychopharmacology (Berlin).

[CR203] Pigott HE, Leventhal AM, Alter GS, Boren J (2010). Efficacy and effectiveness of antidepressants: current status of research. Psychother Psychosom.

[CR204] Pitzalis MV, Iacoviello M, Todarello O, Fioretti A, Guida P, Massari F, Mastropasqua F, Russo GD, Rizzon P (2001). Depression but not anxiety influences the autonomic control of heart rate after myocardial infarction. Am Heart J.

[CR205] Porsolt R, Brossard G, Hautbois C, Roux S (2001). Rodent models of depression: forced swimming and tail suspension behavioral despair tests in rats and mice. Curr Protoc Neurosci.

[CR206] Pucilowski O, Overstreet D, Rezvani A (1993). Chronic mild stress-induced anhedonia: greater effect in a genetic rat model of depression. Physiol Behav.

[CR207] Quan MN, Zheng CG, Zhang N, Han DD, Tian YT, Zhang T, Yang Z (2011). Impairments of behavior, information flow between thalamus and cortex, and prefrontal cortical synaptic plasticity in an animal model of depression. Brain Res Bull.

[CR208] Qui BS, Mei QB, Liu L, Tchou-Wong KM (2004). Effects of nitric oxide on gastric ulceration induced by nicotine and cold-restraint stress. World J Gastroenterol.

[CR209] Raab A, Dantzer R, Michaud B, Mormede P, Taghzouti K, Simon H, Le M (1986). Behavioral, physiological and immunological consequences of social status and aggression in chronically coexisting stress-intruder dyads of male rats. Physiol Behav.

[CR210] Rao RT, Androulakis IP (2020). Modeling inter-sex and inter-individual variability in response to chronopharmacological administration of synthetic glucocorticoids. Chronobiol Int.

[CR211] Raya J, Girardi C, Esumi L, Ferreira L, Hipólide D (2018). Multiple trial inhibitory avoidance acquisition and retrieval are resistant to chronic stress. Behav Process.

[CR212] Reid I, Forbes N, Stewart C, Matthews K (1997). Chronic mild stress and depressive disorder: a useful new model?. Psychopharmacology (Berlin).

[CR213] Remus JL, Stewart LT, Camp RM, Novak CM, Johnson JD (2015). Interaction of Metabolic Stress With Chronic Mild Stress in Altering Brain Cytokines and Sucrose Preference. Behav Neurosci.

[CR214] Riga D, Theijs JT, De Vries TJ, Smit AB, Spijker S (2015). Social defeat-induced anhedonia: effects on operant sucrose-seeking behavior. Front Behav Neurosci.

[CR215] Rizvi SJ, Pizzagalli DA, Sproule BA, Kennedy SH (2016). Assessing anhedonia in depression: potentials and pitfalls. Neurosci Biobehav Rev.

[CR216] Robinson JH (2009). Colony variability under the spotlight in animal models of arthritis. Arthritis Res Ther.

[CR217] Rüedi-Bettschen D, Zhang W, Russig H, Ferger B, Weston A, Pedersen EM (2006). Early deprivation leads to altered behavioural, autonomic and endocrine responses to environmental challenge in adult Fischer rats. Eur J Neurosci.

[CR218] Safer DJ, Zito JM (2019). Short- and Long-Term Antidepressant Clinical Trials for Major Depressive Disorder in Youth: Findings and Concerns. Front Psychiatry.

[CR219] Sampogna G, Del Vecchio V, Giallonardo V, Luciano M, Fiorillo A (2020). Diagnosis, clinical features, and therapeutic implications of agitated depression. Psychiatr Clin North Am.

[CR220] Santangeli O, Lehtikuja H, Palomäki E, Wigren H, Paunio T, Porkka-Heiskanen T (2016). Sleep and behavior in cross-fostering rats: developmental and sex aspects. Sleep..

[CR221] Scheggi S, De Montis MG, Gambarana C (2018). Making Sense of Rodent Models of Anhedonia. Int J Neuropsychopharmacol.

[CR222] Scherholz M, Rao R, Androulakis I (2020). Modeling inter-sex and inter-individual variability in response to chronopharmacological administration of synthetic glucocorticoids. Chronobiol Int.

[CR223] Schmidt M, Scharf S, Sterlemann V, Ganea K, Liebl C, Holsboer F, Müller MB (2010). High susceptibility to chronic social stress is associated with a depression-like phenotype. Psychoneuroendocrinology..

[CR224] Schoenecker B, Heller KE, Freimanis T (2000). Development of stereotypies and polydipsia in wild caught bank voles (Clethrionomys glareolus) and their laboratory-bred offspring. Is polydipsia a symptom of diabetes mellitus?. Appl Anim Behav Sci.

[CR225] Schweizer MC, Henniger MS, Sillaber (2009). Chronic mild stress (CMS) in mice: of anhedonia, 'anomalous anxiolysis' and activity. PLoS One.

[CR226] Shaham Y, Klein L, Alvares K, Grunberg N (1993). Effects of stress on oral fentanyl consumption in rats in an operant self-administration paradigm. Pharmacol Biochem Behav.

[CR227] Shen J, Xu LL, Qu CJ, Sun HM, Zhang JJ (2018). Resveratrol prevents cognitive deficits induced by chronic unpredictable mild stress: Sirt1/miR-134 signalling pathway regulates CREB/BDNF expression in hippocampus in vivo and in vitro. Behav Brain Res.

[CR228] Silva R, Mesquita AR, Bessa J, Sousa JC, Sotiropoulos I, Leao P, Almeida OFX, Sousa N (2008). Lithium blocks stress-induced changes in depressive-like behavior and hippocampal cell fate: the role of glycogen-synthase-kinase-3 beta. Neuroscience.

[CR229] Slattery DA, Cryan JF (2014). The ups and downs of modelling mood disorders in rodents. ILAR J.

[CR230] Slattery DA, Cryan JF (2017). Modelling depression in animals: at the interface of reward and stress pathways. Psychopharmacol..

[CR231] Slattery D, Markou A, Cryan J (2007). Evaluation of reward processes in an animal model of depression. Psychopharmacology (Berlin).

[CR232] Slattery DA, Uschold N, Magoni M, Bär J, Popoli M, Neumann ID, Reber SO (2012). Behavioural consequences of two chronic psychosocial stress paradigms: anxiety without depression. Psychoneuroendocrinology..

[CR233] Soblosky J, Thurmond J (1986). Biochemical and behavioral correlates of chronic stress: effects of tricyclic antidepressants. Pharmacol Biochem Behav.

[CR234] Song YC, Sun RX, Ji ZY, Li XX, Fu Q, Ma SP (2018). Perilla aldehyde attenuates CUMS-induced depressive-like behaviors via regulating TXNIP/TRX/NLRP3 pathway in rats. Life Sci.

[CR235] Spasojevic N, Stefanovic B, Jovanovic P, Dronjak S (2016). Anxiety and hyperlocomotion induced by chronic unpredictable mild stress can be moderated with melatonin. Treatment Folia Biol (Praha).

[CR236] Steimer T, Driscoll P (2005). Inter-individual vs line/strain differences in psychogenetically selected Roman High-(RHA) and Low-(RLA) Avoidance rats: neuroendocrine and behavioural aspects. Neurosci Biobehav Rev.

[CR237] Stephan FK, Zucker I (1972). Circadian rhythms in drinking behavior and locomotor activity of rats are eliminated by hypothalamic lesions. Proc Natl Acad Sci U S A.

[CR238] Sterlemann V, Rammes G, Wolf M, Liebl C, Ganea K, Müller MB, Schmidt MV (2010). Chronic social stress during adolescence induces cognitive impairment in aged mice. Hippocampus..

[CR239] Stevenson JR, McMahon EK, Boner W, Haussmann MF (2019). Oxytocin administration prevents cellular aging caused by social isolation. Psychoneuroendocrinology.

[CR240] Stockton MD, Whitney G (1974). Effects of genotype, sugar, and concentration on sugar preference of laboratory mice (Mus musculus). J Comp Physiol Psychol.

[CR241] Strekalova TV (1995). The characteristics of the defensive behavior of rats in accordance with their resistance to emotional stress. Zh Vyssh Nerv Deiat Im I P Pavlova.

[CR242] Strekalova T, Kalueff A, Laporte J (2008). Optimization of the chronic stress depression model in C57 BL/6 mice: evidences for improved validity. Behavioral models in stress research.

[CR243] Strekalova T (2021) How the sucrose preference succeeds or fails as a measurement of anhedonia. Springer Protocols, Ed. by J. Harro, pp 1–9 (in press)

[CR244] Strekalova T, Steinbusch H, Gould T (2009). Factors of reproducibility of stress-induced anhedonia in chronic stress depression models in mice. Mood and Anxiety related phenotypes in mice: characterization using behavioral tests.

[CR245] Strekalova T, Steinbusch H (2010). Measuring behavior with chronic stress depression model in mice. Prog Neuropsychopharmacol Biol Psychiatry.

[CR246] Strekalova T, Spanagel R, Bartsch D, Henn F, Gass P (2004). Stressed-induced anhedonia in mice is associated with deficits in forced swimming and exploration. Neuropsychopharm..

[CR247] Strekalova T, Spanagel R, Dolgov O, Bartsch D (2005). Stress-induced hyperlocomotion as a confounding factor in anxiety and depression models in mice. Behav Pharmacol.

[CR248] Strekalova T, Gorenkova N, Schunk E, Dolgov O, Bartsch D (2006). Selective effects of citalopram in the mouse model of stress-induced anhedonia with control effects for chronic stress. Behav Pharmacol.

[CR249] Strekalova T, Cespuglio R, Kovalson V (2009). Sleep structure during chronic stress and anhedonia in the mouse model of depression. Behavioral Models in Stress Research.

[CR250] Strekalova T, Couch Y, Kholod N, Boyks M, Malin D, Leprince P, Steinbusch H (2011). Update in the methodology of the chronic stress paradigm: internal control matters. Behav Brain Funct.

[CR251] Strekalova T, Costa-Nunes J, Veniaminova E, Kubatiev A, Lesch K, Chekhonin V, Evans M, Steinbusch H (2016). Insulin receptor sensitizer, dicholine succinate, prevents both Toll-like receptor 4 (TLR4) upregulation and affective changes induced by a high-cholesterol diet in mice. J Affect Disord.

[CR252] Sun HL, Su RJ, Zhang XX, Wen J, Yao D, Gao XR, Zhu Z, Li H (2017). Hippocampal GR- and CB1-mediated mGluR5 differentially produces susceptibility and resilience to acute and chronic mild stress in rats. Neuroscience..

[CR253] Taliaz D, Loya A, Gersner R, Haramati S, Chen A, Zangen A (2011). Resilience to chronic stress is mediated by hippocampal brain-derived neurotrophic factor. J Neurosci.

[CR254] Tang M, Huang H, Li S, Zhou M, Liu Z, Huang R, Liao W, Xie P, Zhou J (2019). Hippocampal proteomic changes of susceptibility and resilience to depression or anxiety in a rat model of chronic mild stress. Transl Psychiatry.

[CR255] Theilmann W, Kleimann A, Rhein M, Bleich S, Frieling H, Löscher W, Brandt C (2016). Behavioral differences of male Wistar rats from different vendors in vulnerability and resilience to chronic mild stress are reflected in epigenetic regulation and expression of p11. Brain Res.

[CR256] Theilmann W, Rosenholm M, Hampel P, Loscher W, Rantamaki T (2020) Lack of antidepressant effects of burst-suppressing isoflurane anesthesia in adult male Wistar outbred rats subjected to chronic mild stress. PLoS One:15 10.1371/journal.pone.0235046 PMC7313995 32579566

[CR257] Tonissaar M, Herm L, Rinken A, Harro J (2006). Individual differences in sucrose intake and preference in the rat: circadian variation and association with dopamine D2 receptor function in striatum and nucleus accumbens. Neurosci Lett.

[CR258] Trajkova S, d'Errico A, Soffietti R, Sacerdote C, Ricceri F (2019). Use of antidepressants and risk of incident stroke: a systematic review and meta-analysis neuroepidemiology.

[CR259] Ulrich-Lai YM, Figueiredo HF, Ostrander MM, Choi DC, Engeland WC, Herman JP (2006). Chronic stress induces adrenal hyperplasia and hypertrophy in a subregion-specific manner. Am J Physiol Endocrinol Metab.

[CR260] Unal G, Canbeyli R (2019). Psychomotor retardation in depression: A critical measure of the forced swim test. Behav Brain Res.

[CR261] Ushijma K, Morikawa T, Higuchi S, Ohdo S, To H (2006). Chronobiological disturbances with hyperthermia and hypercortisolism induced by chronic mild stress in rats. Behav Brain Res.

[CR262] Vitale G, Ruggieri V, Filaferro M, Frigeri C, Alboni S, Tascedda F (2009). Chronic treatment with the selective NOP receptor antagonist [Nphe 1, Arg 14, Lys 15]N/OFQ-NH 2 (UFP-101) reverses the behavioural and biochemical effects of unpredictable chronic mild stress in rats. Psychopharmacology (Berl)..

[CR263] Von Frijtag J, Reijmers L, Van der Harst J, Leus I, Van den Bos R, Spruijt B (2000). Defeat followed by individual housing results in long-term impaired reward- and cognition-related behaviours in rats. Behav Brain Res.

[CR264] Wainwright SR, Workman JL, Tehrani A, Hamson DK, Chow C, Lieblich SE, Galea LA (2016). Testosterone has antidepressant-like efficacy and facilitates imipramine-induced neuroplasticity in male rats exposed to chronic unpredictable stress. Horm Behav.

[CR265] Wang CH, Zhang XL, Li Y, Wang GD, Wang XK, Dong J, Ning QF (2015). Role of hippocampus mitogen-activated protein kinase phosphatase-1 mRNA expression and DNA methylation in the depression of the rats with chronic unpredicted stress. Cell Mol Neurobiol.

[CR266] Wang YQ, Li R, Zhang MQ, Zhang Z, Qu WM, Huang ZL (2015). The neurobiological mechanisms and treatments of REM sleep disturbances in depression. Curr Neuropharmacol.

[CR267] Wang YL, Han QQ, Gong WQ, Pan DH, Wang LZ, Hu W, Yang M, Li B, Yu J, Liu Q (2018) Microglial activation mediates chronic mild stress-induced depressive- and anxiety-like behavior in adult rats. J Neuroinflammation 15 10.1186/s12974-018-1054-3 PMC5773028 29343269

[CR268] Wang Y, Liu D, Li X, Liu Y, Wu Y (2021). Antidepressants use and the risk of type 2 diabetes mellitus: A systematic review and meta-analysis. J Affect Disord.

[CR269] Watkins LL, Grossman P (1999). Association of depressive symptoms with reduced baroreflex cardiac control in coronary artery disease. Am Heart J.

[CR270] Wegener G, Mathe A, Neumann I (2012). Selectively bred rodents as models of depression and anxiety. Curr Top Behav Neurosci.

[CR271] Weiss JM (1997). Does decreased sucrose intake indicate loss of preference in CMS model?. Psychopharmacology.

[CR272] Weiss JM, Simson PG (1986). Depression in an animal model: focus on the locus ceruleus. CIBA Found Symp.

[CR273] Weiss IC, Di Iorio L, Feldon J, Domeney AM (2000). Strain differences in the isolation-induced effects on prepulse inhibition of the acoustic startle response and on locomotor activity. Behav Neurosci.

[CR274] Willner P (1992). Chronic mild stress-induced anhedonia: a realistic animal model of depression. Neurosci Biobehav Rev.

[CR275] Willner P (2005). Chronic mild stress (CMS) revisited: consistency and behavioural-neurobiological concordance in the effects of CMS. Neuropsych..

[CR276] Willner P (2016). Reliability of the chronic mild stress model of depression: a user survey. Neurobiol Stress.

[CR277] Willner P (2017). The chronic mild stress (CMS) model of depression: history, evaluation and usage. Neurobiol Stress.

[CR278] Willner P, Belzung C (2015). Treatment-resistant depression: are animal models of depression fit for purpose?. Psychopharmacology.

[CR279] Willner P, Towell D, Sampson S, Sophokleous R, Muscat (1987). Reduction of sucrose preference by chronic unpredictable mild stress, and its restoration by a tricyclic antidepressant. Psychopharmacology (Berlin).

[CR280] Wind T, Rijkeboer M, Andersson G, Riper H (2020). The COVID-19 pandemic: The 'black swan' for mental health care and a turning point for e-health. Internet Interv.

[CR281] World Health Organization (2012) https://www.who.int/mental_health/management/depression/wfmh_paper_depression_wmhd_2012.pdf

[CR282] World Health Organization (2017). Depression and Other Common Mental Disorders: Global Health Estimates.

[CR283] Yang L, Shi LJ, Tang B, Han QQ, Yu J, Wu GC (2016). Opposite Sex Contact and Isolation: A Novel Depression/Anxiety Model. Neurosci Bull.

[CR284] Yankelevitch-Yahav R, Franko M, Huly A, Doron R (2015) The forced swim test as a model of depressive-like behavior. J Vis Exp 97 10.3791/52587 PMC4401172 25867960

[CR285] Yu ZL, Chen N, Hu D, Chen WX, Yuan Y, Meng SQ et al (2020) Decreased density of perineuronal net in prelimbic cortex is linked to depressive-like behavior in young-aged rats. Front Mol Neurosci 13 10.3389/fnmol.2020.00004 PMC7025547 32116542

[CR286] Yue N, Huang HJ, Zhu XC, Han QQ, Wang YL, Li B, Liu Q, Wu GC, Zhang YQ, Yu J (2017) Activation of P2X7 receptor and NLRP3 inflammasome assembly in hippocampal glial cells mediates chronic stress-induced depressive like behaviors. J Neuroinflammation 14 10.1186/s12974-017-0865-y PMC5424302 28486969

[CR287] Zhan C, Kalueff AV, Song C (2019). Minocycline ameliorates anxiety-related self-grooming behaviors and alters hippocampal neuroinflammation, GABA and serum cholesterol levels in female Sprague-Dawley rats subjected to chronic unpredictable mild stress. Behav Brain Res.

[CR288] Zhang M, Li R, Wang Y, Huang Z (2017). Neural plasticity is involved in physiological sleep, depressive sleep disturbances, and antidepressant treatments. Neural Plasticity in Mood Disorders.

[CR289] Zhang YQ, Yuan S, Pu JC, Yang LN, Zhou XY, Liu LX, Jiang XF, Zhang HP, Teng T, Tian L, Xie P (2018). Integrated metabolomics and proteomics analysis of Hippocampus in a rat model of depression. Neuroscience.

[CR290] Zhang YN, Zhang XL, Liu N, Ren SY, Xia CY, Yang X et al (2021) Comparative proteomic characterization of ventral hippocampus in susceptible and resilient rats subjected to chronic unpredictable stress. Front Neurosci 15 10.3389/fnins.2021.675430 PMC8249003 34220431

[CR291] Zorkina YA, Zubkov EA, Morozova AY, Ushakova VM, Chekhonin VP (2019). The comparison of a new ultrasound-induced depression model to the chronic mild stress paradigm. Front Behav Neurosci.

[CR292] Zurawek D, Kusmider M, Faron-Gorecka A, Gruca P, Pabian P, Solich J, Kolasa M, Papp M, Dziedzicka-Wasylewska M (2017). Reciprocal MicroRNA expression in mesocortical circuit and its interplay with serotonin transporter define resilient rats in the chronic mild stress. Mol Neurobiol.

[CR293] Zurawek D, Gruca P, Antkiewicz-Michaluk L, Dziedzicka-Wasylewska M (2019). Resilient phenotype in chronic mild stress paradigm is associated with altered expression levels of miR-18a-5p and serotonin 5-HT1a receptor in dorsal part of the hippocampus. Mol Neurobiol.

[CR294] Zuzarte P, Duong A, Figueira M, Vitali A, Scola G (2018). Current therapeutic approaches for targeting inflammation in depression and cardiovascular disease. Curr Drug Metab.

