# Supplementary material for: Chronic mild stress paradigm as a rat model of depression: facts, artifacts, and future perspectives
Source: Psychopharmacology (Berl). 2022 Jan 24;239(3):663–93. doi: 10.1007/s00213-021-05982-w (PMC8785013; doi:10.1007/s00213-021-05982-w)
Supplement: Supplementary file 1 — (DOCX 262 kb) [file 213_2021_5982_MOESM1_ESM.docx]

# Supplementary File 1

#
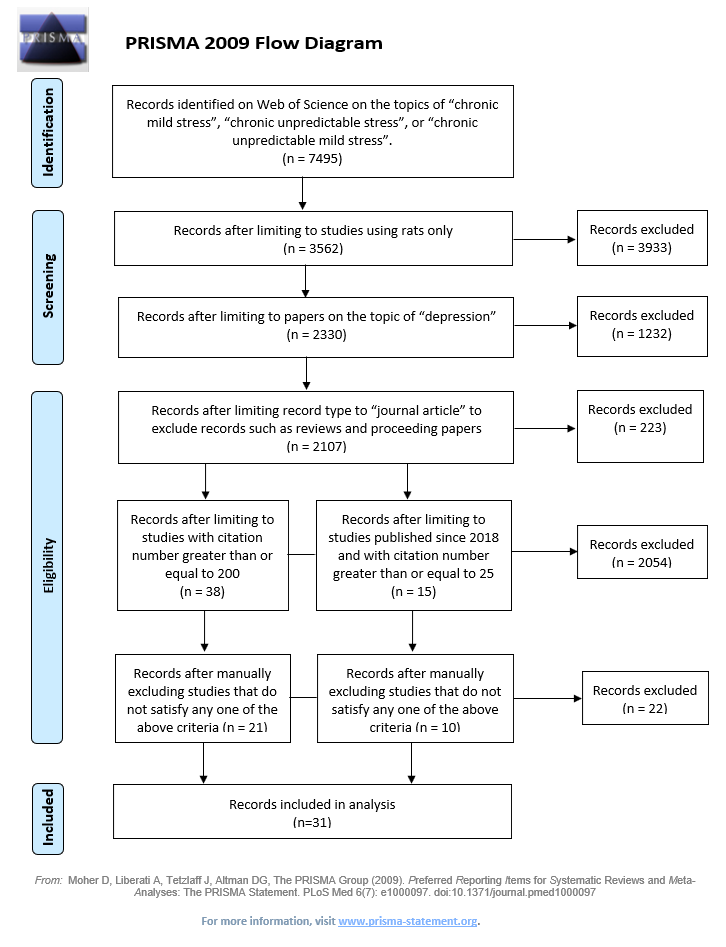


**Table S1. Literature search pipeline utilized for Table 1 and 2.** The search of literature for Tables 1 and 2 was conducted as described in the main text (*see the text*).


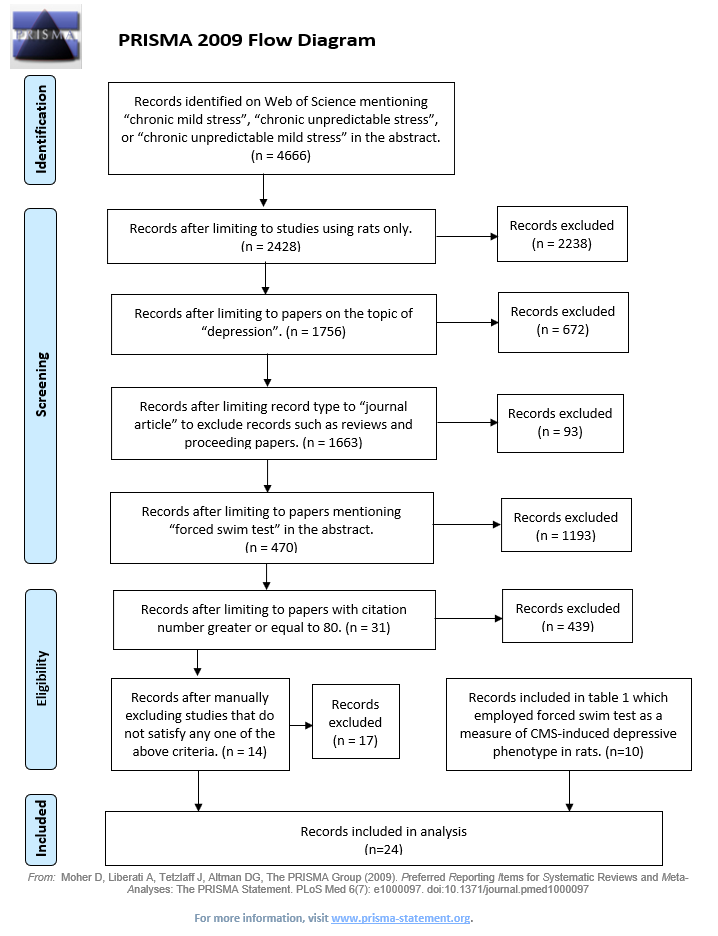


**Table S2. Literature search pipeline utilized to review Table 3.**


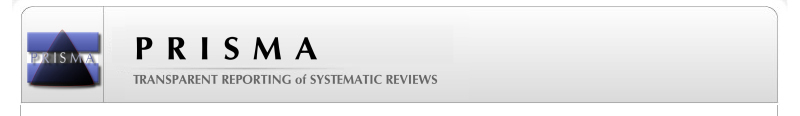
**PRISMA 2009 Flow Diagram**

## Screening

## Eligibility

Records excluded
(n = 8)

Records excluded
(n = 1)

Records excluded
(n = 1)

Records after manually excluding any papers that do not satisfy any of the above criteria.

(n = 17)

Records after limiting language in English only

(n = 25)

Records after limiting record type to “journal article” to exclude records such as reviews and proceeding papers

(n = 26)

Records after limiting to papers included both the terms “susceptible” and “resilient” only

(n = 27)

## Included

Records included in analysis

(n=17)

Records excluded
(n = 2401)

Records excluded
(n = 1263)

Records after limiting to papers on the topic of “depression”

(n = 2428)

(n = )

Records after limiting to studies using rats only
(n = 3691)

Records excluded
(n = 4147)

## Identification

Records identified on Web of Science on the topics of “chronic mild stress”, “chronic unpredictable stress”, or “chronic unpredictable mild stress”.
(n = 7838)

**Table S3. Literature search pipeline utilized to review Table 5.**

# Table legends for Supplementary File 2

**Table S1. Results of WoS search for main Tables 1 and 2.** Papers on the CMS model that reported used stressors, behavioral tests, and depressive-like changes (as of Mar 7, 2021) were utilized. Papers that were included in the present review are marked blue; papers that did not meet the search criteria and therefore were removed from analysis are marked gray. A subset of the relevant papers found was also used to review the literature on SCT/SPT employed in assessing the CMS model. The table includes articles’ details, as well as journals’ details and citations data.

**Table S2. Results of WoS search for the main Table 3.** Papers with reports of FST application for assessing the CMS model (as of Mar 13, 2021) were analyzed. Papers that were included in the present review are marked blue; papers that did not meet the search criteria and therefore were removed from analysis are marked gray.The table includes articles’ details, as well as journals’ details and citations data.

**Table S3. Results of WoS search for the main Table 5.** Papers with reports of stratification of CMS-rats to ‘resilient’ and ‘susceptible’ phenotypes upon anhedonic features in the sucrose test the CMS model (as of Jul 15, 2021) were analyzed. Papers that were included in the present review are marked blue; papers that did not meet the search criteria and therefore were removed from analysis are marked gray. The table includes articles’ details, as well as journals’ details and citations data.

# References

Bai, M., Zhu, X.Z., Zhang, Y., Zhang, S., Zhang, L., Xue, L., Yi, J.Y., Yao, S.Q., Zhang, X.W., 2012. Abnormal Hippocampal BDNF and miR-16 Expression Is Associated with Depression-Like Behaviors Induced by Stress during Early Life. Plos One 7. <https://doi.org/10.1371/journal.pone.0046921>

Banasr, M., Chowdhury, G.M.I., Terwilliger, R., Newton, S.S., Duman, R.S., Behar, K.L., Sanacora, G., 2010. Glial pathology in an animal model of depression: reversal of stress-induced cellular, metabolic and behavioral deficits by the glutamate-modulating drug riluzole. Molecular Psychiatry 15, 501–511. <https://doi.org/10.1038/mp.2008.106>

Banasr, M., Duman, R.S., 2008. Glial Loss in the Prefrontal Cortex Is Sufficient to Induce Depressive-like Behaviors. Biological Psychiatry 64, 863–870. <https://doi.org/10.1016/j.biopsych.2008.06.008>

Banasr, M., Valentine, G.W., Li, X.Y., Gourley, S.L., Taylor, J.R., Duman, R.S., 2007. Chronic unpredictable stress decreases cell proliferation in the cerebral cortex of the adult rat. Biological Psychiatry 62, 496–504. <https://doi.org/10.1016/j.biopsych.2007.02.006>

Bekris, S., Antoniou, K., Daskas, S., Papadopoulou-Daifoti, Z., 2005. Behavioural and neurochemical effects induced by chronic mild stress applied to two different rat strains. Behavioural Brain Research 161, 45–59. <https://doi.org/10.1016/j.bbr.2005.01.005>

Bessa, J.M., Ferreira, D., Melo, I., Marques, F., Cerqueira, J.J., Palha, J.A., Almeida, O.F.X., Sousa, N., 2009a. The mood-improving actions of antidepressants do not depend on neurogenesis but are associated with neuronal remodeling. Molecular Psychiatry 14, 764–773. <https://doi.org/10.1038/mp.2008.119>

Bessa, J.M., Mesquita, A.R., Oliveira, M., Pego, J.M., Cerqueira, J.J., Palha, J.A., Almeida, O.F.X., Sousa, N., 2009b. A trans-dimensional approach to the behavioral aspects of depression. Frontiers in Behavioral Neuroscience 3. <https://doi.org/10.3389/neuro.08.001.2009>

Beyer, C.E., Dwyer, J.M., Piesla, M.J., Platt, B.J., Shen, R., Rahman, Z., Chan, K., Manners, M.T., Samad, T.A., Kennedy, J.D., Bingham, B., Whiteside, G.T., 2010. Depression-like phenotype following chronic CB1 receptor antagonism. Neurobiology of Disease 39, 148–155. <https://doi.org/10.1016/j.nbd.2010.03.020>

Bondi, C.O., Rodriguez, G., Gould, G.G., Frazer, A., Morilak, D.A., 2008. Chronic unpredictable stress induces a cognitive deficit and anxiety-like behavior in rats that is prevented by chronic antidepressant drug treatment. Neuropsychopharmacology 33, 320–331. <https://doi.org/10.1038/sj.npp.1301410>

Bortolato, M., Mangieri, R.A., Fu, J., Kim, J.H., Arguello, O., Duranti, A., Tontini, A., Mor, M., Tarzia, G., Piomelli, D., 2007. Antidepressant-like activity of the fatty acid amide hydrolase inhibitor URB597 in a rat model of chronic mild stress. Biological Psychiatry 62, 1103–1110. <https://doi.org/10.1016/j.biopsych.2006.12.001>

Chang, C.H., Grace, A.A., 2014. Amygdala-Ventral Pallidum Pathway Decreases Dopamine Activity After Chronic Mild Stress in Rats. Biological Psychiatry 76, 223–230. <https://doi.org/10.1016/j.biopsych.2013.09.020>

Cheeta, S., Ruigt, G., vanProosdij, J., Willner, P., 1997. Changes in sleep architecture following chronic mild stress. Biological Psychiatry 41, 419–427. <https://doi.org/10.1016/s0006-3223(96)00058-3>

Christiansen, S.L., Hojgaard, K., Wiborg, O., Bouzinova, E.V., 2016. Disturbed diurnal rhythm of three classical phase markers in the chronic mild stress rat model of depression. Neuroscience Research 110, 43–48. <https://doi.org/10.1016/j.neures.2016.03.002>

Cudnoch-Jedrzejewska, A., Szczepanska-Sadowska, E., Dobruch, J., Gomolka, R., Puchalska, L., 2010. Brain vasopressin V-1 receptors contribute to enhanced cardiovascular responses to acute stress in chronically stressed rats and rats with myocardial infarcton. American Journal of Physiology-Regulatory Integrative and Comparative Physiology 298, R672–R680. <https://doi.org/10.1152/ajpregu.00543.2009>

Czeh, B., Vardya, I., Varga, Z., Febbraro, F., Csabai, D., Martis, L.S., Hoigaard, K., Henningsen, K., Bouzinova, E.V., Miseta, A., Jensen, K., Wiborg, O., 2018. Long-Term Stress Disrupts the Structural and Functional Integrity of GABAergic Neuronal Networks in the Medial Prefrontal Cortex of Rats. Frontiers in Cellular Neuroscience 12. <https://doi.org/10.3389/fncel.2018.00148>

Dalla, C., Antoniou, K., Drossopoulou, G., Xagoraris, M., Kokras, N., Sfikakis, A., Papadopoulou-Daifoti, Z., 2005. Chronic mild stress impact: Are females more vulnerable? Neuroscience 135, 703–714. <https://doi.org/10.1016/j.neuroscience.2005.06.068>

Daquila, P.S., Brain, P., Willner, P., 1994. EFFECTS OF CHRONIC MILD STRESS ON PERFORMANCE IN BEHAVIORAL-TESTS RELEVANT TO ANXIETY AND DEPRESSION. Physiology & Behavior 56, 861–867. <https://doi.org/10.1016/0031-9384(94)90316-6>

Fan, C.Q., Song, Q.Q., Wang, P., Li, Y., Yang, M., Yu, S.Y., 2018. Neuroprotective Effects of Ginsenoside-Rg1 Against Depression-Like Behaviors via Suppressing Glial Activation, Synaptic Deficits, and Neuronal Apoptosis in Rats. Frontiers in Immunology 9. <https://doi.org/10.3389/fimmu.2018.02889>

Franklin, T.C., Wohleb, E.S., Zhang, Y., Fogaca, M., Hare, B., Duman, R.S., 2018. Persistent Increase in Microglial RAGE Contributes to Chronic Stress-Induced Priming of Depressive-like Behavior. Biological Psychiatry 83, 50–60. <https://doi.org/10.1016/j.biopsych.2017.06.034>

Garza, J.C., Guo, M., Zhang, W., Lu, X.Y., 2012. Leptin restores adult hippocampal neurogenesis in a chronic unpredictable stress model of depression and reverses glucocorticoid-induced inhibition of GSK-3 beta/beta-catenin signaling. Molecular Psychiatry 17, 790–808. <https://doi.org/10.1038/mp.2011.161>

Greene, J., Banasr, M., Lee, B., Warner-Schmidt, J., Duman, R.S., 2009. Vascular Endothelial Growth Factor Signaling is Required for the Behavioral Actions of Antidepressant Treatment: Pharmacological and Cellular Characterization. Neuropsychopharmacology 34, 2459–2468. <https://doi.org/10.1038/npp.2009.68>

Grippo, A.J., Moffitt, J.A., Johnson, A.K., 2008. Evaluation of baroreceptor reflex function in the chronic mild stress rodent model of depression. Psychosomatic Medicine 70, 435–443. <https://doi.org/10.1097/PSY.0b013e31816ff7dd>

Grippo, A.J., Moffitt, J.A., Johnson, A.K., 2002. Cardiovascular alterations and autonomic imbalance in an experimental model of depression. American Journal of Physiology-Regulatory Integrative and Comparative Physiology 282, R1333–R1341. <https://doi.org/10.1152/ajpregu.00614.2001>

Grippo, A.J., Santos, C.M., Johnson, R.F., Beltz, T.G., Martins, J.B., Felder, R.B., Johnson, A.K., 2004. Increased susceptibility to ventricular arrhythmias in a rodent model of experimental depression. American Journal of Physiology-Heart and Circulatory Physiology 286, H619–H626. <https://doi.org/10.1152/ajpheart.00450.2003>

Grønli, J., Dagestad, G., Milde, A.M., Murison, R., Bramham, C.R., 2012. Post-transcriptional effects and interactions between chronic mild stress and acute sleep deprivation: Regulation of translation factor and cytoplasmic polyadenylation element-binding protein phosphorylation. Behav Brain Res 235, 251–262. <https://doi.org/10.1016/j.bbr.2012.08.008>

Gronli, J., Murison, R., Bjorvatn, B., Sorensen, E., Portas, C.M., Ursin, R., 2004. Chronic mild stress affects sucrose intake and sleep in rats. Behavioural Brain Research 150, 139–147. <https://doi.org/10.1016/s0166-4328(03)00252-3>

Hao, Z.K., Wang, W., Guo, R., Liu, H., 2019. Faecalibacteriun prausnitzii (ATCC 27766) has preventive and therapeutic effects on chronic unpredictable mild stress-induced depression-like and anxiety-like behavior in rats. Psychoneuroendocrinology 104, 132–142. <https://doi.org/10.1016/j.psyneuen.2019.02.025>

Heine, V.M., Maslam, S., Zareno, J., Joels, M., Lucassen, P.J., 2004. Suppressed proliferation and apoptotic changes in the rat dentate gyrus after acute and chronic stress are reversible. European Journal of Neuroscience 19, 131–144. <https://doi.org/10.1046/j.1460-9568.2003.03100.x>

Hill, M.N., Patel, S., Carrier, E.J., Rademacher, D.J., Ormerod, B.K., Hillard, C.J., Gorzalka, B.B., 2005. Downregulation of endocannabinoid signaling in the hippocampus following chronic unpredictable stress. Neuropsychopharmacology 30, 508–515. <https://doi.org/10.1038/sj.npp.1300601>

Jayatissa, M.N., Bisgaard, C., Tingstrom, A., Papp, M., Wiborg, O., 2006. Hippocampal cytogenesis correlates to escitalopram-mediated recovery in a chronic mild stress rat model of depression. Neuropsychopharmacology 31, 2395–2404. <https://doi.org/10.1038/sj.npp.1301041>

Karson, A., Demirtas, T., Bayramgurler, D., Balci, F., Utkan, T., 2013. Chronic Administration of Infliximab (TNF-alpha Inhibitor) Decreases Depression and Anxiety-like Behaviour in Rat Model of Chronic Mild Stress. Basic & Clinical Pharmacology & Toxicology 112, 335–340. <https://doi.org/10.1111/bcpt.12037>

Koo, J.W., Duman, R.S., 2008. IL-1 beta is an essential mediator of the antineurogenic and anhedonic effects of stress. Proceedings of the National Academy of Sciences of the United States of America 105, 751–756. <https://doi.org/10.1073/pnas.0708092105>

Kreisel, T., Frank, M.G., Licht, T., Reshef, R., Ben-Menachem-Zidon, O., Baratta, M.V., Maier, S.F., Yirmiya, R., 2014. Dynamic microglial alterations underlie stress-induced depressive-like behavior and suppressed neurogenesis. Molecular Psychiatry 19, 699–709. <https://doi.org/10.1038/mp.2013.155>

Larsen, M.H., Mikkelsen, J.D., Hay-Schmidt, A., Sandi, C., 2010. Regulation of brain-derived neurotrophic factor (BDNF) in the chronic unpredictable stress rat model and the effects of chronic antidepressant treatment. Journal of Psychiatric Research 44, 808–816. <https://doi.org/10.1016/j.jpsychires.2010.01.005>

Li, N.X., Liu, R.J., Dwyer, J.M., Banasr, M., Lee, B., Son, H., Li, X.Y., Aghajanian, G., Duman, R.S., 2011. Glutamate N-methyl-D-aspartate Receptor Antagonists Rapidly Reverse Behavioral and Synaptic Deficits Caused by Chronic Stress Exposure. Biological Psychiatry 69, 754–761. <https://doi.org/10.1016/j.biopsych.2010.12.015>

Li, S.X., Han, Y., Xu, L.Z., Yuan, K., Zhang, R.X., Sun, C.Y., Xu, D.F., Yuan, M., Deng, J.H., Meng, S.Q., Gao, X.J., Wen, Q., Liu, L.J., Zhu, W.L., Xue, Y.X., Zhao, M., Shi, J., Lu, L., 2018. Uncoupling DAPK1 from NMDA receptor GluN2B subunit exerts rapid antidepressant-like effects. Molecular Psychiatry 23, 597–608. <https://doi.org/10.1038/mp.2017.85>

Liu, B., Xu, C., Wu, X., Liu, F., Du, Y., Sun, J., Tao, J., Dong, J., 2015. ICARIIN EXERTS AN ANTIDEPRESSANT EFFECT IN AN UNPREDICTABLE CHRONIC MILD STRESS MODEL OF DEPRESSION IN RATS AND IS ASSOCIATED WITH THE REGULATION OF HIPPOCAMPAL NEUROINFLAMMATION. Neuroscience 294, 193–205. <https://doi.org/10.1016/j.neuroscience.2015.02.053>

Lu, X.Y., Kim, C.S., Frazer, A., Zhang, W., 2006. Leptin: A potential novel antidepressant. Proceedings of the National Academy of Sciences of the United States of America 103, 1593–1598. <https://doi.org/10.1073/pnas.0508901103>

Lu, Y.X., Ho, C.S., McIntyre, R.S., Wang, W., Ho, R.C., 2018. Effects of vortioxetine and fluoxetine on the level of Brain Derived Neurotrophic Factors (BDNF) in the hippocampus of chronic unpredictable mild stress-induced depressive rats. Brain Research Bulletin 142, 1–7. <https://doi.org/10.1016/j.brainresbull.2018.06.007>

Lucas, G., Rymar, V.V., Du, J., Mnie-Filali, O., Bisgaard, C., Manta, S., Lambas-Senas, L., Wiborg, O., Haddjeri, N., Pineyro, G., Sadikot, A.F., Debonnel, G., 2007. Serotonin(4) (5-HT4) receptor Agonists are putative antidepressants with a rapid onset of action. Neuron 55, 712–725. <https://doi.org/10.1016/j.neuron.2007.07.041>

Luo, D.D., An, S.C., Zhang, X., 2008. Involvement of hippocampal serotonin and neuropeptide Y in depression induced by chronic unpredicted mild stress. Brain Research Bulletin 77, 8–12. <https://doi.org/10.1016/j.brainresbull.2008.05.010>

Matchkov, V.V., Kravtsova, V.V., Wiborg, O., Aalkjaer, C., Bouzinova, E.V., 2015. Chronic selective serotonin reuptake inhibition modulates endothelial dysfunction and oxidative state in rat chronic mild stress model of depression. American Journal of Physiology-Regulatory Integrative and Comparative Physiology 309, R814–R823. <https://doi.org/10.1152/ajpregu.00337.2014>

Moreau, J.L., Scherschlicht, R., Jenck, F., Martin, J.R., 1995. CHRONIC MILD STRESS-INDUCED ANHEDONIA MODEL OF DEPRESSION - SLEEP ABNORMALITIES AND CURATIVE EFFECTS OF ELECTROSHOCK TREATMENT. Behavioural Pharmacology 6, 682–687.

Papp, M., Gruca, P., Boyer, P.A., Mocaer, E., 2003. Effect of agomelatine in the chronic mild stress model of depression in the rat. Neuropsychopharmacology 28, 694–703. <https://doi.org/10.1038/sj.npp.1300091>

Papp, M., Moryl, E., 1994. ANTIDEPRESSANT ACTIVITY OF NONCOMPETITIVE AND COMPETITIVE NMDA RECEPTOR ANTAGONISTS IN A CHRONIC MILD STRESS MODEL OF DEPRESSION. European Journal of Pharmacology 263, 1–7. <https://doi.org/10.1016/0014-2999(94)90516-9>

Papp, M., Willner, P., Muscat, R., 1991. AN ANIMAL-MODEL OF ANHEDONIA - ATTENUATION OF SUCROSE CONSUMPTION AND PLACE PREFERENCE CONDITIONING BY CHRONIC UNPREDICTABLE MILD STRESS. Psychopharmacology 104, 255–259. <https://doi.org/10.1007/bf02244188>

Park, S.E., Park, D., Song, K.I., Seong, J.K., Chung, S., Youn, I., 2017. Differential heart rate variability and physiological responses associated with accumulated short- and long-term stress in rodents. Physiology & Behavior 171, 21–31. <https://doi.org/10.1016/j.physbeh.2016.12.036>

Quan, M.N., Zheng, C.G., Zhang, N., Han, D.D., Tian, Y.T., Zhang, T., Yang, Z., 2011. Impairments of behavior, information flow between thalamus and cortex, and prefrontal cortical synaptic plasticity in an animal model of depression. Brain Research Bulletin 85, 109–116. <https://doi.org/10.1016/j.brainresbull.2011.03.002>

Shen, J., Xu, L.L., Qu, C.J., Sun, H.M., Zhang, J.J., 2018. Resveratrol prevents cognitive deficits induced by chronic unpredictable mild stress: Sirt1/miR-134 signalling pathway regulates CREB/BDNF expression in hippocampus in vivo and in vitro. Behavioural Brain Research 349, 1–7. <https://doi.org/10.1016/j.bbr.2018.04.050>

Silva, R., Mesquita, A.R., Bessa, J., Sousa, J.C., Sotiropoulos, I., Leao, P., Almeida, O.F.X., Sousa, N., 2008. Lithium blocks stress-induced changes in depressive-like behavior and hippocampal cell fate: The role of glycogen-synthase-kinase-3 beta. Neuroscience 152, 656–669. <https://doi.org/10.1016/j.neuroscience.2007.12.026>

Song, Y.C., Sun, R.X., Ji, Z.Y., Li, X.X., Fu, Q., Ma, S.P., 2018. Perilla aldehyde attenuates CUMS-induced depressive-like behaviors via regulating TXNIP/TRX/NLRP3 pathway in rats. Life Sciences 206, 117–124. <https://doi.org/10.1016/j.lfs.2018.05.038>

Stemmelin, J., Cohen, C., Terranova, J.P., Lopez-Grancha, M., Pichat, P., Bergis, O., Decobert, M., Santucci, V., Francon, D., Alonso, R., Stahl, S.M., Keane, P., Avenet, P., Scatton, B., le Fur, G., Griebel, G., 2008. Stimulation of the beta(3)-adrenoceptor as a novel treatment strategy for anxiety and depressive disorders. Neuropsychopharmacology 33, 574–587. <https://doi.org/10.1038/sj.npp.1301424>

Ulrich-Lai, Y.M., Figueiredo, H.F., Ostrander, M.M., Choi, D.C., Engeland, W.C., Herman, J.P., 2006. Chronic stress induces adrenal hyperplasia and hypertrophy in a subregion-specific manner. American Journal of Physiology-Endocrinology and Metabolism 291, E965–E973. <https://doi.org/10.1152/ajpendo.00070.2006>

Ushijima, K., Morikawa, T., To, H., Higuchi, S., Ohdo, S., 2006. Chronobiological disturbances with hyperthermia and hypercortisolism induced by chronic mild stress in rats. Behavioural Brain Research 173, 326–330. <https://doi.org/10.1016/j.bbr.2006.06.038>

Wang, D., An, S.C., Zhang, X., 2008. Prevention of chronic stress-induced depression-like behavior by inducible nitric oxide inhibitor. Neuroscience Letters 433, 59–64. <https://doi.org/10.1016/j.neulet.2007.12.041>

Wang, Y.L., Han, Q.Q., Gong, W.Q., Pan, D.H., Wang, L.Z., Hu, W., Yang, M., Li, B., Yu, J., Liu, Q., 2018. Microglial activation mediates chronic mild stress-induced depressive- and anxiety-like behavior in adult rats. Journal of Neuroinflammation 15. <https://doi.org/10.1186/s12974-018-1054-3>

Yang, L.M., Hu, B., Xia, Y.H., Zhang, B.L., Zhao, H., 2008. Lateral habenula lesions improve the behavioral response in depressed rats via increasing the serotonin level in dorsal raphe nucleus. Behavioural Brain Research 188, 84–90. <https://doi.org/10.1016/j.bbr.2007.10.022>

Yue, N., Huang, H.J., Zhu, X.C., Han, Q.Q., Wang, Y.L., Li, B., Liu, Q., Wu, G.C., Zhang, Y.Q., Yu, J., 2017. Activation of P2X7 receptor and NLRP3 inflammasome assembly in hippocampal glial cells mediates chronic stress-induced depressive-like behaviors. Journal of Neuroinflammation 14. <https://doi.org/10.1186/s12974-017-0865-y>

Zhang, Y.Q., Yuan, S., Pu, J.C., Yang, L.N., Zhou, X.Y., Liu, L.X., Jiang, X.F., Zhang, H.P., Teng, T., Tian, L., Xie, P., 2018. Integrated Metabolomics and Proteomics Analysis of Hippocampus in a Rat Model of Depression. Neuroscience 371, 207–220. <https://doi.org/10.1016/j.neuroscience.2017.12.001>
