# Supplementary material for: Chronic mild stress paradigm as a rat model of depression: facts, artifacts, and future perspectives
Source: Psychopharmacology (Berl). 2022 Jan 24;239(3):663–93. doi: 10.1007/s00213-021-05982-w (PMC8785013; doi:10.1007/s00213-021-05982-w)
Supplement: Supplementary file 3 — (PDF 928 kb) [file 213_2021_5982_MOESM3_ESM.pdf]

Mathematic comparison of variability of sucrose intake and sucrose preference

A comparison of variability of sucrose intake (a) and sucrose preference (b), where the latter is calculated by the formula:

Sucrose preference (b)= Sucrose intake (a) / Sucrose intake (a) + Water intake x 100%

results to a conclusion as for smaller values of variances (Var) for sucrose preference than for sucrose intake. Since water intake in CMS procedure was reported to change in various directions, i.e. increase or decrease in a course of stress, as well as total liquid intake (see ms text), these two parameters can be regarded as independent variables.

Approximations for mean and variance of a ratio

Let us consider random variables a and b having support $[0, \infty)$ and assume that $g(a, b) = a/(a + b)$. Then, we find approximations for $E(g)$ and $Var(g)$ by using the first and second Taylor expansions of $g(a, b)$. For any $f(x, y)$, the bivariate first order Taylor expansion about any $\theta = (\theta_x, \theta_y)$ is

$$f(x,y) = f(\theta) + f_x^{'}(\theta)(x - \theta_x) + f_y^{'}(\theta)(y - \theta_y) + R \quad (1)$$

where R is a remainder of smaller order than terms in the equation. Let us denote the means of a and b as μ_a and μ_b, respectively.

For our example where $g(a, b) = a/(a + b)$, the approximation of the mean is $f(\mu_a, \mu_b) = \mu_a/(\mu_a + \mu_b)$. The second order Taylor expansion is

$$f(x,y) = f(\theta) + f_x^{'}(\theta)(x - \theta_x) + f_y^{'}(\theta)(y - \theta_y) + \frac{1}{2}\{f_{xx}^{''}(\theta)(x - \theta_x)^2 + 2f_{xy}^{''}(\theta)(x - \theta_x)(y - \theta_y) + f_{yy}^{''}(\theta)(y - \theta_y)^2\} + R \quad (2)$$

The better approximation for $g(a, b)$ is the expansion around $\theta = (\mu_a, \mu_b)$ defined as follows

$$E(g(a,b)) \approx g(\theta) + \frac{1}{2}\{g_{aa}^{''}(\theta)Var(a) + 2g_{ab}^{''}(\theta)Cov(a,b) + g_{bb}^{''}(\theta)Var(y)\}, \quad (3)$$

where we added the definitions of variance $Var(a) = E[(a - \mu_a)^2]$ and covariance $Cov(a, b) = E[(a - \mu_a)(b - \mu_b)]$ and use the facts that $E(a - \mu_a) = 0$ and $Cov(a, b) = 0$ since the processes a and b are assumed to be independend and non-correlated.

where we added the definitions of variance $Var(a) = E[(a - \mu_a)^2]$ and covariance $Cov(a, b) = E[(a - \mu_a)(b - \mu_b)]$ and use the facts that $E(a - \mu_a) = 0$ and $Cov(a, b) = 0$ since the processes a and b are assumed to be independend and non-correlated.

For the ratio $g(a, b) = a/(a + b)$, we get the following final result for the mean approximation

$$E(g(a,b)) \approx \frac{\mu_a}{(\mu_a + \mu_b)} + \frac{1}{(\mu_a + \mu_b)^3}\{Var(b) - Var(a)\} \quad (4)$$

By the definition of variance, the variance of $g(a, b)$ is

$$Var(a, b) = E\{[g(a, b) - E(g(a, b))]^2\} \tag{5}$$

and then using from above $E(g(a, b)) \approx g(\mu_a, \mu_b)$

$$Var(a, b) = E\{[g(a, b) - g(\mu_a, \mu_b)]^2\} \tag{6}$$

We finally get the variance of $g(a, b)$ defined as

$$Var(a, b) = \frac{\mu_b^2}{(\mu_a + \mu_b)^4} Var(a) + \frac{\mu_a^2}{(\mu_a + \mu_b)^4} Var(b) \tag{7}$$

As can be seen from the last equation (7), the resulting values of variance $Var(a, b)$ have to be smaller than those of either $Var(a)$ or $Var(b)$.

References:

Kendall's Advanced Theory of Statistics, Arnold, London, 1998, 6th Edition, Volume 1, by Stuart and Ord, p. 351.

Survival Models and Data Analysis, John Wiley and Sons NY, 1980, by Elandt-Johnson and Johnson, p. 69.

Let us consider random variables a and b having support $[0, \infty)$ and assume that $g(a, b) = a/(a + b)$. Then, we find approximations for $E(g)$ and $Var(g)$ by using the first and second Taylor expansions of $g(a, b)$. For any $f(x, y)$, the bivariate first order Taylor expansion about any $\theta = (\theta_x, \theta_y)$ is

$$f(x, y) = f(\theta) + f_x^{'}(\theta)(x - \theta_x) + f_y^{'}(\theta)(y - \theta_y) + R \tag{1}$$

where R is a remainder of smaller order than terms in the equation. Let us denote the means of a and b as μ_a and μ_b, respectively.

For our example where $g(a, b) = a/(a + b)$, the approximation of the mean is $f(\mu_a, \mu_b) = \mu_a/(\mu_a + \mu_b)$. The second order Taylor expansion is

$$\begin{aligned} f(x, y) = f(\theta) &+ f_x^{'}(\theta)(x - \theta_x) + f_y^{'}(\theta)(y - \theta_y) \\ &+ \frac{1}{2}\{f_{xx}^{''}(\theta)(x - \theta_x)^2 + 2f_{xy}^{''}(\theta)(x - \theta_x)(y - \theta_y) + f_{yy}^{''}(\theta)(y - \theta_y)^2\} + R \end{aligned} \tag{2}$$

The better approximation for $g(a, b)$ is the expansion around $\theta = (\mu_a, \mu_b)$ defined as follows

$$E(g(a, b)) \approx g(\theta) + \frac{1}{2}\{g_{aa}^{''}(\theta)Var(a) + 2g_{ab}^{''}(\theta)Cov(a, b) + g_{bb}^{''}(\theta)Var(y)\}, \tag{3}$$

where we added the definitions of variance $Var(a) = E[(a - \mu_a)^2]$ and covariance $Cov(a, b) = E[(a - \mu_a)(b - \mu_b)]$ and use the facts that $E(a - \mu_a) = 0$ and $Cov(a, b) = 0$ since the processes a and b are assumed to be independend and non-correlated.

Approximations for mean and variance of a ratio

Let us consider random variables a and b having support $[0, \infty)$ and assume that $g(a, b) = a/(a+b)$. Then, we find approximations for $E(g)$ and $Var(g)$ by using the first and second Taylor expansions of $g(a, b)$. For any $f(x, y)$, the bivariate first order Taylor expansion about any $\theta = (\theta_x, \theta_y)$ is

$$f(x,y) = f(\theta) + f_x^{'}(\theta)(x - \theta_x) + f_y^{'}(\theta)(y - \theta_y) + R \tag{1}$$

where R is a remainder of smaller order than terms in the equation. Let us denote the means of a and b as μ_a and μ_b, respectively.

For our example where $g(a, b) = a/(a+b)$, the approximation of the mean is $f(\mu_a, \mu_b) = \mu_a/(\mu_a + \mu_b)$. The second order Taylor expansion is

$$\begin{aligned} f(x,y) = {} & f(\theta) + f_x^{'}(\theta)(x - \theta_x) + f_y^{'}(\theta)(y - \theta_y) \\ & + \frac{1}{2}\{f_{xx}^{''}(\theta)(x-\theta_x)^2 + 2f_{xy}^{''}(\theta)(x-\theta_x)(y-\theta_y) + f_{yy}^{''}(\theta)(y-\theta_y)^2\} + R \end{aligned} \tag{2}$$

The better approximation for $g(a, b)$ is the expansion around $\theta = (\mu_a, \mu_b)$ defined as follows

$$E(g(a,b)) \approx g(\theta) + \frac{1}{2}\{g_{aa}^{''}(\theta)Var(a) + 2g_{ab}^{''}(\theta)Cov(a,b) + g_{bb}^{''}(\theta)Var(y)\}, \tag{3}$$

where we added the definitions of variance $Var(a) = E[(a - \mu_a)^2]$ and covariance $Cov(a, b) = E[(a - \mu_a)(b - \mu_b)]$ and use the facts that $E(a - \mu_a) = 0$ and $Cov(a, b) = 0$ since the processes a and b are assumed to be independend and non-correlated.
For the ratio $g(a, b) = a/(a + b)$, we get the following final result for the mean approximation

$$E(g(a,b)) \approx \frac{\mu_a}{(\mu_a + \mu_b)} + \frac{1}{(\mu_a+\mu_b)^3}\{Var(b) - Var(a)\} \tag{4}$$

By the definition of variance, the variance of $g(a, b)$ is

$$Var(a,b) = E\{[g(a,b) - E(g(a,b))]^2\} \tag{5}$$

and then using from above $E(g(a, b)) \approx g(\mu_a, \mu_b)$

$$Var(a,b) = E\{[g(a,b) - g(\mu_a, \mu_b)]^2\} \tag{6}$$

We finally get the variance of $g(a, b)$ defined as

$$Var(a,b) = \frac{\mu_b^2}{(\mu_a+\mu_b)^4}Var(a) + \frac{\mu_a^2}{(\mu_a+\mu_b)^4}Var(b) \tag{7}$$

As can be seen from the last equation (7), the resulting values of variance $Var(a, b)$ have to be smaller than those of either $Var(a)$ or $Var(b)$.
